# Supplementary material for: Activation of IRF3 in cardiomyocytes impairs mitochondrial oxidative function through PGC-1α inhibition and drives heart failure
Source: Nat Commun. 2026 Feb 27;17:2051. doi: 10.1038/s41467-026-69792-4 (PMC12948977; doi:10.1038/s41467-026-69792-4)
Supplement: Supplementary file 1 — Supplementary Information [file 41467_2026_69792_MOESM1_ESM.pdf]

Supplemental Data

Table of Contents

1. Supplementary Figure Legends **1**

2. Supplementary Tables and Legends........ ………………………………….……………..... **10**

3. Supplementary Figures……………….……………………………………………………….…...**12**

4. Lipidomics check list

1. Supplementary Figure Legends

Fig. S1: Type I IFN signaling alterations in ischemic cardiomyopathy and inflamed cardiomyocytes.

a Expression of *IRF3* target genes regulating type I IFN signaling in the left ventricular tissue of humans with ICM compared to NF hearts determined by qPCR. NF (n=7) and ICM patients (n=10), biological replicates. Statistical significance was calculated by unpaired two-tailed Student's t test. *P* values are shown on the bar graph.

b Single cell analysis showing gene expression of *IRF3* in various cell types in patients with heart failure compared to healthy non-failing control hearts.

c Myocardial infarction was induced in 14wk old C57BL6 mice by LAD ligation. Cardiomyocytes were isolated and subjected to cell fractionation. Immunoblot showing pIRF3 and IRF3 levels in the cytoplasm and nuclear fraction of cardiomyocytes. GAPDH and Histone H3 were used to detect cytoplasmic and nuclear purity. n=2 per sample, biological replicates.

d Cardiomyocytes isolated from 12wk old wild-type mice treated with 1mg/kg LPS for 2h and gene expression of *Irf3* and its target genes regulating type I IFN signaling was determined by qPCR. Each data point indicates cardiomyocytes isolated from one C57bl/6 mouse. n=3 replicates per group, significance was calculated by unpaired two-tailed Student's t test. *P* values are shown on the bar graph.

e Cardiomyocytes isolated from neonatal rat ventricular tissue was treated with 0.5ug/ml polyinosinic:polycytidylic acid (Poly I:C) for 6h and gene expression of *Irf3* and its target genes regulating type I IFN signaling was determined by qPCR. n=6 replicates per group. Statistical significance was calculated by unpaired two-tailed Student's t test. *P* values are shown next to the bar graph.

f Gene expression of IRF3 target genes by qPCR in cardiomyocytes isolated from neonatal rat ventricular tissue after treatment with 1µg/ml mitochondrial DNA (mtDNA) isolated from left ventricle of 16wk old Wistar rats. n=4 replicates per group. Statistical significance was calculated by unpaired two-tailed Student's t test. *P* values are shown next to the bar graph.
All data are represented as mean ± SEM. Source data file is provided.

Fig. S2: Effect of IRF3 deficiency in the cardiomyocytes of mice with ischemic cardiomyopathy and NRCMs.

a Relative *Irf3* mRNA levels in the liver, epididymal white adipose tissue and gastrocnemius muscle of CMI3KO mice compared to αMHC-Cre upon LAD determined by qPCR, Cre (n=5), CMI3KO (n=4), biological replicates.

b Relative *Irf3* mRNA levels in isolated cardiac fibroblasts, cardiac endothelial cells and other cells (supernatant pellet after cardiomyocytes and fibroblasts isolation) from CMI3KO and αMHC-Cre mice, n=4(fibroblasts), n=3(endothelial cells), n=3(other cells) .

c Body weight of male CMI3KO and αMHC-Cre mice upon LAD. Sham: n=7(αMHC-Cre), n=8(CMI3KO); LAD: n=7(αMHC-Cre), n=9(CMI3KO), biological replicates.

d Left ventricular posterior wall end systole analyzed by echocardiography in CMI3KO and αMHC-Cre mice upon LAD. Analyzed by one-way ANOVA with Šídák's multiple comparison test, n=8 (αMHC-Cre), n=8(CMI3KO). Cre-Sham vs. Cre-LAD: ****P*=0.0005; Cre-LAD vs. CMI3KO-LAD:**P*=0.0288.

e-f siRNA mediated knockdown of IRF3 in neonatal rat cardiomyocytes at mRNA (N=6 per group) and protein level (n=3 per group). mRNA knockdown: ****P*=6.6X10^{-5}; protein knockdown: ****P*=0006.

g Expression of IRF family members in neonatal rat cardiomyocytes upon siRNA mediated knockdown of *Irf3*, n=6 per group. *Irf3*: ****P*=0.00015, *Irf7*: ***P*=0.00311.

h Expression of *Irf3* target inflammatory genes in neonatal rat cardiomyocytes upon siRNA mediated knockdown of *Irf3* in the absence and presence LPS treatment, n=6 replicates per group. *P* values are shown next to the bar graph.

All data are represented as mean ± SEM. Statistical significance was assessed by unpaired two-tailed Student's t test (**e**, **f**, **g**, **h**). Source data file is provided.

Fig. S3: Muscle-specific activation of IRF3 results in impaired cardiac function.

a Representative model showing generation strategy of MI3OE transgenic mouse.

b Gross morphology of control (Cre^+) and MI3OE mice at 4 weeks age.

c Body weight measured in control (Cre^+) and MI3OE mice, n=7 per group. ***P*=0.0017.

d Kaplan Meier plot showing survival curve of control (Cre^+) and MI3OE mice.

e Gene expression of *Irf3* and its target genes in left ventricular tissue determined by qPCR, n=4 per group. *Irf3*: ***P*=0.0020; *Ifnβ:* ***P*=0.0055; *Ifit1:* ****P*=$1.9X10^{-5}$; *Ccl5*: ***P*=0.0055.

f Gene expression of *Irf3* and its target genes in gastrocnemius tissue determined by qPCR, n=4 per group. *Irf3*: ****P*=.$3.8X10^{-5}$; *Ifnβ:* **P*=0.0582; *Ifit1:* ****P*=0.0010; *Ccl2*: ***P*=0.0025; *Ccl5:* **P*=0.0191.

g Immunoblot showing IRF3 protein levels in cardiac and gastrocnemius muscle, n=4 per group.

h-j Plasma glucose, insulin and CCL2 levels determined using glucometer, RIA and ELISA kit, respectively. Glucose: ***P*=0.0110; Insulin: ***P*=0.0096; CCL2: ****P*= 0.0001.

k-l Heart and lung weight to body weight ratio measured in the control (Cre^+) and MI3OE mice, n=7 per group. ***P*= 0.0019.

m Representative images from echocardiography performed in awake 4 week old control (Cre^+) and MI3OE mice.

n-p Cardiac function determined by echocardiography in awake 4 week old control (Cre^+) and MI3OE mice. Cre (n=5), MI3OE (n=3), biological replicates. %EF: ****P*=0.0011; %FS: ***P*=0.0028; %LVPWs: **P*=0.0194.

Survival curve, tissue weight and echocardiography experiments were performed using independent cohorts.

All data are represented as mean ± SEM. Statistical significance was calculated by unpaired two-tailed Student's t test. Source data file is provided.

Fig. S4: Effect on inflammatory and mitochondrial oxidative pathways upon IRF3 activation in the cardiomyocytes of CMI3OE mice.

a Venn diagram showing number of differentially regulated genes in the left ventricle of CMI3OE compared to Cre control.

b Volcano plot showing differentially regulated genes in the left ventricle of CMI3OE compared to Cre control.

c GO pathway analysis using differentially upregulated genes in the left ventricle of CMI3OE.

d GO pathway analysis using differentially downregulated genes in the left ventricle of CMI3OE.

e Masson's trichrome IHC in CMI3OE cardiac tissue compared to Cre control.

f Immunoblot showing expression of αSMA in CMI3OE compared to Cre control in the left ventricular tissue. N=4 per group, biological replicates.

Fig. S5: Effect on inflammation and mitochondrial marker genes upon IRF3-2D expression in cardiomyocytes.

a Immunoblot showing co-immunoprecipitation assay in NRCMs stimulated with Poly I:C (2µg/ml) for 4h. Immunoprecipitation was performed with anti-PGC-1α antibody

b Immunoblot showing cell fractionation and expression of IRF3 in the cytoplasm, membrane and nuclear fraction of adult cardiomyocytes isolated from CMI3OE mice compared to Cre control. GAPDH, VDAC and Histone H3 were used to detect cytoplasmic, membrane and nuclear purity. n=2 per group, biological replicates.

c Quantification of IRF3 and PGC-1α pooled protein levels in the immunoblot shown in Fig. S5b from the cytoplasm, membrane, and nuclear fractions of cardiomyocytes isolated from CMI3OE mice. IRF3: ***P*=0.0025; PGC-1α: ***P*=0.0039

d Immunoblot showing expression of IRF3-2D in neonatal rat cardiomyocytes (NRCMs).

e Quantification of the immunoblot shown in Fig. S5d. N=3 per group. ****P*=0.0014.

f Immunofluorescence images showing cellular localization of IRF3 upon expression of wild-type IRF3 or IRF3-2D in NRCMs compared to LacZ control.

g Expression of mitochondrial biogenesis marker genes in NRCMs upon IRF3-2D expression. n=6 per group. *Nrf1*: ****P*=0.0002; *Ppargc1α*: ****P*=0.0003.

h Expression of IRF3 target genes in NRCMs by qPCR upon IRF3-2D expression. LacZ (n=6), IRF3-2D (n=5). *Ifit3*: ****P*=$1.9 X 10^{-8}$; *Isg15*: ****P*=$2.8 X 10^{-9}$; *OasII*: ****P*=$3.5 X 10^{-8}$; *Rsad2*: ****P*=$1.5 X 10^{-6}$.

i Gene expression of mitochondrial marker genes in NRCMs upon IRF3-2D expression. LacZ (n=6), IRF3-2D (n=5). *Atp6*: ***P*=0.0036; *Atp8*: ***P*=0.0032[9]; *Cytb*: ****P*=0.0003; *Cox1:* ****P*=0.0010; *Cox2*: ***P*=0.0031; *Cox3*: ***P*=0.0044.

All data are represented as mean ± SEM. Statistical significance was calculated by unpaired two-tailed Student's t test. Source data file is provided.

Fig. S6: Effect on inflammation and mitochondrial marker genes upon PGC-1α expression in cardiomyocytes.

a Expression of IRF3 target genes by qPCR in LPS treated NRCMs upon PGC-1α expression. n=4 per group. *P* values are shown next to the bar graph.

b Expression of mitochondrial oxidative phosphorylation marker genes by qPCR in LPS treated NRCMs upon PGC-1α expression. n=4 per group. *P* values are shown next to the bar graph.

c Expression of IRF3 target genes by qPCR in NRCMs expressing PGC-1α and subjected to hypoxia. N=5 per group. *P* values are shown next to the bar graph.

d Expression of mitochondrial oxidative phosphorylation marker genes by qPCR in NRCMs expressing PGC-1α and subjected to hypoxia. n=5 per group. *P* values are shown next to the bar graph.

e Expression of PPARα target genes in the left ventricle of CMI3OE mice. n=4 per group. *P* values are shown next to the bar graph.

All data are represented as mean ± SEM. Statistical significance was calculated by unpaired two-tailed Student's t test. Source data file is provided.

Fig. S7: Cardiac metabolic alterations and adaptions upon IRF3 activation in cardiomyocytes.

a Volcano plot showing the water soluble metabolites in CMI3OE compared to αMHCMCM mice cardiac tissue.

b Heatmap showing top 30 water soluble metabolites altered in CMI3OE compared to αMHCMCM mice cardiac tissue. Cre (n=6), CMI3OE (n=7).

c Glucose uptake using ^{3}H-deoxyglucose in adult cardiomyocytes isolated from 12 wk old CMI3OE and Cre control mice. Cardiomyocytes were isolated from n=3 per group and each mouse cardiomyocytes were seeded in two 12 well plates. N=6wells/group. ****P*=2X10$^{-6.}$

d Volcano plot showing lipid metabolites in CMI3OE compared to αMHCMCM mice cardiac tissue.

e Heatmap showing top 25 lipid metabolites altered in CMI3OE compared to αMHCMCM mice cardiac tissue. Cre (n=6), CMI3OE (n=7).

f Lipidomic analysis showing relative fold change in plasma lipid class concentration analyzed on Lipidyzer in 12 wk old male CMI3OE mice compared to control mice. Ctrl (n=7), CMI3OE (n=7) are biological replicates. FFA: ***P*=0.0082; HCER: **P*=0.0279; LCER: ***P*=0.0019; SM: ****P*=0.0003.

All data are represented as mean ± SEM. Statistical significance was calculated by unpaired two-tailed Student's t test. Source data file is provided.

Fig. S8: Gene expression alterations in the left ventricle of CMI3OE mice.

a Gene expression of *Bdh1* in the left ventricle of CMI3OE mice compared to αMHCMCM. N=4 per group. **P*=0.0227.

b Gene expression of *Sdha* in the left ventricle of CMI3OE mice compared to αMHCMCM. N=4 per group. ***P=5.9X10^{-6}.

c Heatmap showing differential expression of NAD dehydrogenase complex assembly marker genes in CMI3OE mice compared to αMHCMCM. N=4 per group.

All data are represented as mean ± SEM. Statistical significance was calculated by unpaired two-tailed Student's t test. Source data file is provided.

Fig. S9: Stable isotope labeling assay in cardiomyocytes isolated from adult CMI3OE mice.

a-h Metabolic flux assay in adult cardiomyocytes isolated from Cre control and CMI3OE mice using U-$^{13}C_6$-Glucose at 10 min for glycolysis pathway and 120min for TCA cycle. Samples group, 0min: Cre (n=4), CMI3OE (n=4); 10min: Cre (n=5), CMI3OE (n=6); 120min: Cre (n=5), CMI3OE (n=4), independent biological replicates. .

i Gene expression analysis showing effect on PPP marker genes mRNA level in the left ventricle of CMI3OE mice compared to Cre control. n=4 per group.

Fig. S10: Cardiomyocyte-specific moderate PGC-1α expression improves cardiac function in adult CMI3OE mice.

a Gene expression of *Ppargc1α* and *Ppargc1β* in the left ventricular tissue of control mice using AAV9-TnT-PGC-1α compared to AAV9-TnT-EGFP determined by qPCR analysis. N=4 per group. *P=0.0170.

b Tissue weight of CMI3OE treated with AAV9-TnT-PGC-1α compared to AAV9-TnT-EGFP controls. n=5 (Ctrl-AAV-EGFP), n=5 (Ctrl-AAV-PGC-1α), n=6 (CMI3OE-AAV-EGFP), n=5 (CMI3OE-AAV-PGC-1α). Spleen: Ctrl-AAV-EGFP vs Ctrl-AAV-PGC-1α, *P=0256; CMI3OE-AAV-EGFP vs CMI3OE-AAV-PGC-1α, *P=0256; Kidney: Ctrl-AAV-EGFP vs CMI3OE-AAV-EGFP, *P=0230.

c-d Left ventricular posterior wall end diastole (LVPWd) and left ventricular posterior wall end systole (LVPWs) measurement in CMI3OE treated with AAV9-TnT-PGC-1α compared to AAV9-TnT-EGFP controls. n=6 (Ctrl-AAV-EGFP), n=5 (Ctrl-AAV-PGC-1α), n=6 (CMI3OE-AAV-EGFP), n=5 (CMI3OE-AAV-PGC-1α), biological replicates.

Fig. S11: PGC-1α expression attenuates cardiac inflammation and upregulates mitochondrial OXPHOS levels in CMI3OE mice.

a Venn diagram showing number of differentially regulated genes in the left ventricle of CMI3OE-AAV-PGC-1α compared to CMI3OE-AAV-EGFP mice.

b Volcano plot showing differentially regulated genes in the left ventricle of CMI3OE-AAV-PGC-1α compared to CMI3OE-AAV-EGFP mice.

c GO pathway analysis using differentially downregulated genes in the left ventricle of CMI3OE-AAV-PGC-1α compared to CMI3OE-AAV-EGFP mice.

d GO pathway analysis using differentially upregulated genes in the left ventricle of CMI3OE-AA-V-PGC-1α compared to CMI3OE-AAV-EGFP mice.

e Differential expression of genes regulating NAD metabolism in the left ventricle of CMI3OE-AAV-PGC-1α compared to CMI3OE-AAV-EGFP mice.

2. Supplementary Tables

Table S1. Plasma samples collected from 12wk old male CMI3OE and αMHCMCM mice were used for the plasma measurements. Data is represented as Mean±SEM. Statistical significance was calculated by unpaired two-tailed Student's t test.

Plasma parameter	αMHCMCM	CMI3OE	*P* value	N
**Glucose (mg/dl)**	159.43±9.94	182.29±10.35	0.1372	Cre (n=7), CMI3OE (n=7)
**FFA (mM)**	0.75±0.12	1.06±0.02*	0.0213	Cre (n=5), CMI3OE (n=7)
**TG (mg/dl)**	108.83±11.77	101.73±8.07	0.6165	Cre (n=5), CMI3OE (n=7)
**Cholesterol (mg/dl)**	69.57±2.32	73.01±2.14	0.3099	Cre (n=5), CMI3OE (n=7)

Table S2: LC Gradient scheme. Separation of NAD/NADH and NADP/NADPH was achieved by the following LC gradient scheme using mobile phase A (50/50; Acetonitrile / Water with 5 mM ammonium acetate + 0.05% (v/v) ammonium hydroxide, pH 10) and mobile phase B (90/10; Acetonitrile : Water with 5 mM ammonium acetate + 0.05% (v/v) ammonium hydroxide, pH 10).

**Time (min)**	**Flow (mL/min)**	**%A**	**%B**
**0**	0,400	5	95
**0,5**	0,400	5	95
**0,51**	0,350	5	95
**5**	0,350	90	10
**5,1**	0,300	90	10
**5,2**	0,300	100	0
**10**	0,300	100	0
**10,3**	0,400	5	95
**15**	0,400	5	95

Fig. S1: Type I IFN signaling alterations in ischemic cardiomyopathy and inflamed cardiomyocytes.

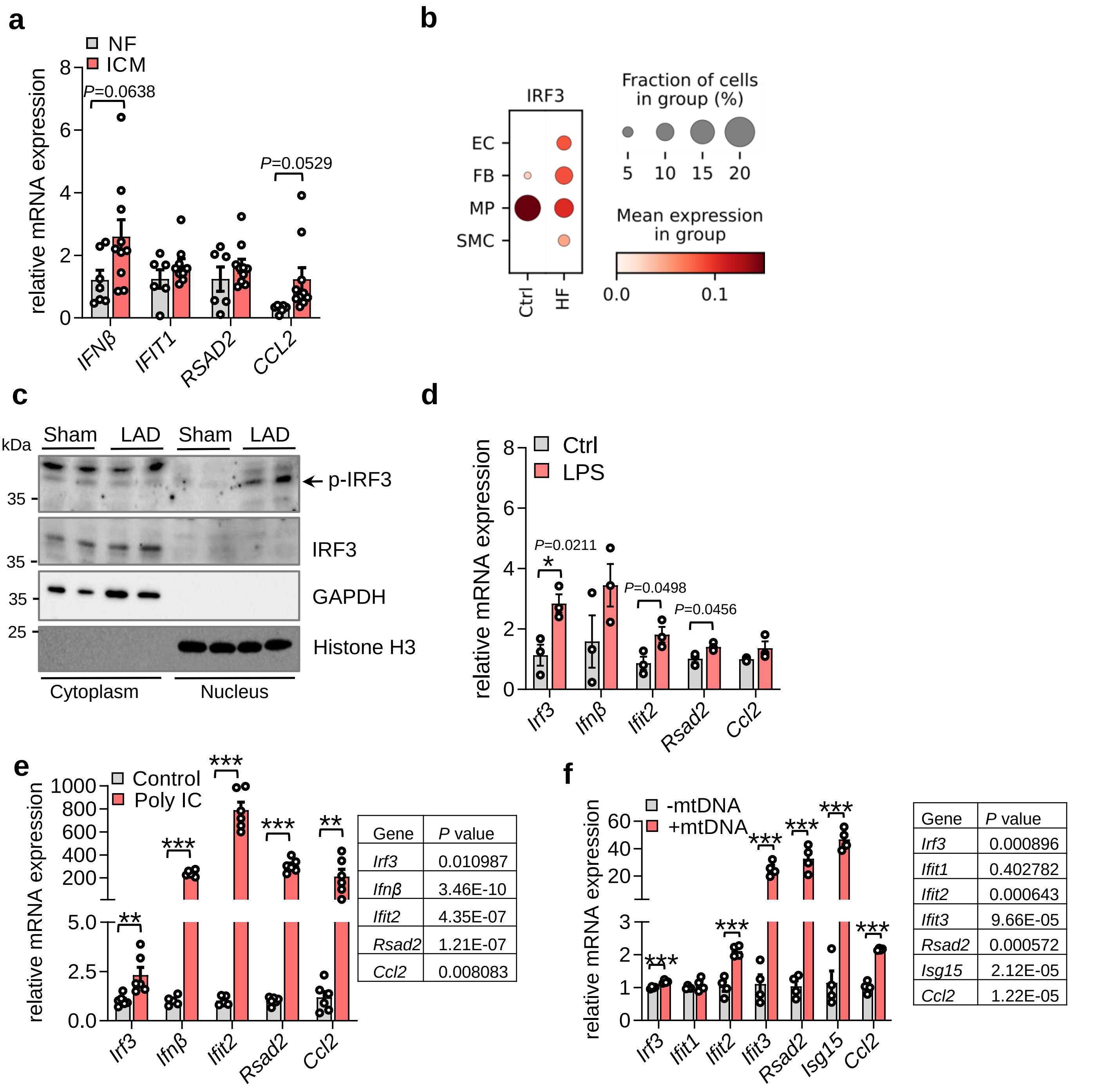

Gene	*P* value
Irf3	0.010987
Ifnβ	3.46E-10
Ifit2	4.35E-07
Rsad2	1.21E-07
Ccl2	0.008083

Gene	*P* value
Irf3	0.000896
Ifit1	0.402782
Ifit2	0.000643
Ifit3	9.66E-05
Rsad2	0.000572
Isg15	2.12E-05
Ccl2	1.22E-05

a Expression of *IRF3* target genes regulating type I IFN signaling in the left ventricular tissue of humans with ICM compared to NF hearts determined by qPCR. NF (n=7) and ICM patients (n=10), biological replicates. Statistical significance was calculated by unpaired two-tailed Student's t test. *P* values are shown on the bar graph.

b Single cell analysis showing gene expression of *IRF3* in various cell types in patients with heart failure compared to healthy non-failing control hearts.

c Myocardial infarction was induced in 14wk old C57BL6 mice by LAD ligation. Cardiomyocytes were isolated and subjected to cell fractionation. Immunoblot showing pIRF3 and IRF3 levels in the cytoplasm and nuclear fraction of cardiomyocytes. GAPDH and Histone H3 were used to detect cytoplasmic and nuclear purity. n=2 per sample, biological replicates.

d Cardiomyocytes isolated from 12wk old wild-type mice treated with 1mg/kg LPS for 2h and gene expression of *Irf3* and its target genes regulating type I IFN signaling was determined by qPCR. Each data point indicates cardiomyocytes isolated from one C57bl/6 mouse. n=3 replicates per group, significance was calculated by unpaired two-tailed Student's t test. *P* values are shown on the bar graph.

e Cardiomyocytes isolated from neonatal rat ventricular tissue was treated with 0.5ug/ml polyinosinic:polycytidylic acid (Poly I:C) for 6h and gene expression of *Irf3* and its target genes regulating type I IFN signaling was determined by qPCR. n=6 replicates per group. Statistical significance was calculated by unpaired two-tailed Student's t test. *P* values are shown next to the bar graph.

f Gene expression of IRF3 target genes by qPCR in cardiomyocytes isolated from neonatal rat ventricular tissue after treatment with 1µg/ml mitochondrial DNA (mtDNA) isolated from left ventricle of 16wk old Wistar rats. n=4 replicates per group. Statistical significance was calculated by unpaired two-tailed Student's t test. *P* values are shown next to the bar graph.

All data are represented as mean ± SEM. Source data file is provided.

Fig. S2: Effect of IRF3 deficiency in the cardiomyocytes of mice with ischemic cardiomyopathy and NRCMs.

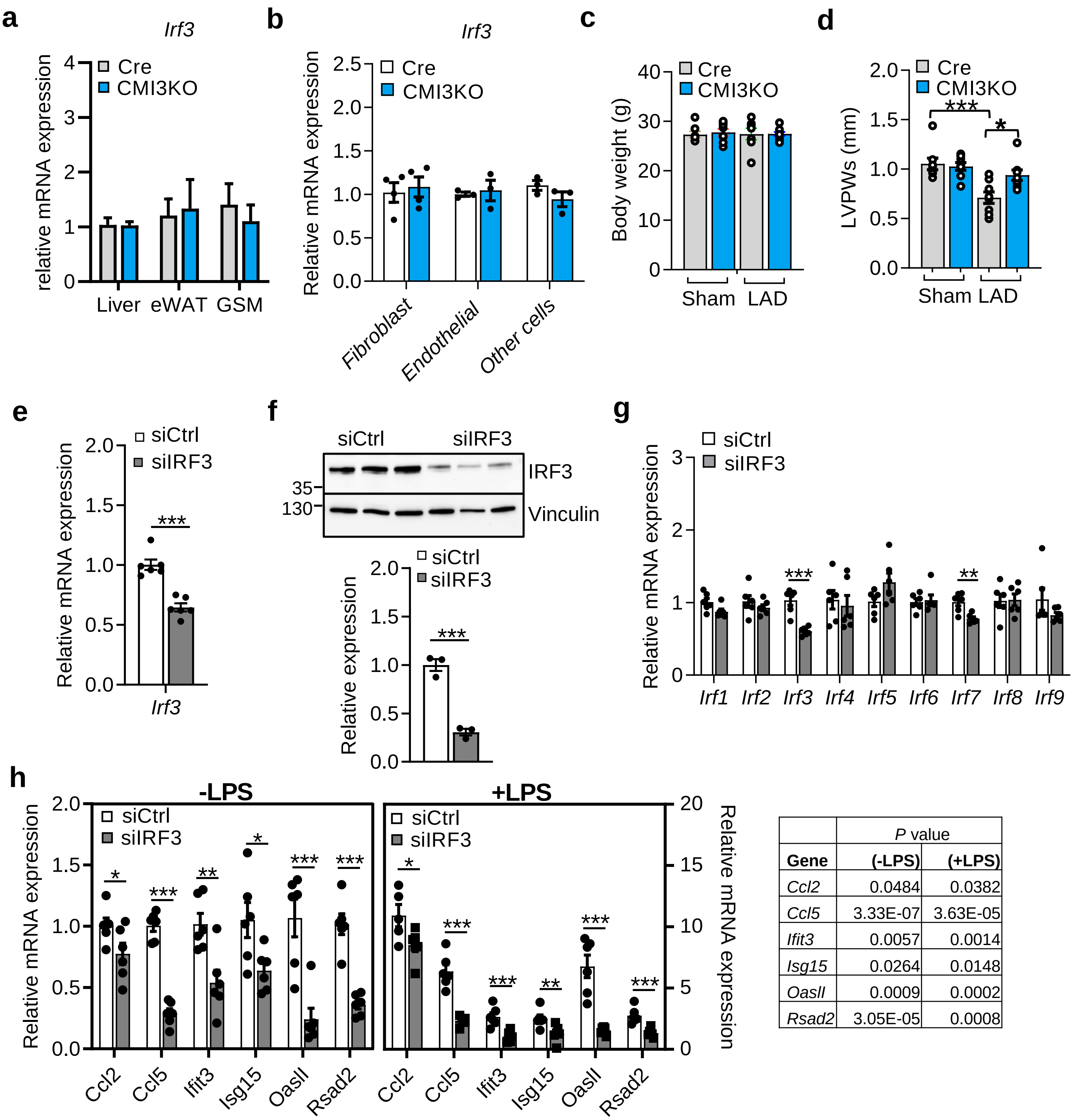

	P value	
**Gene**	**(-LPS)**	**(+LPS)**
Ccl2	0.0484	0.0382
Ccl5	3.33E-07	3.63E-05
Ifit3	0.0057	0.0014
Isg15	0.0264	0.0148
OasII	0.0009	0.0002
Rsad2	3.05E-05	0.0008

a Relative *Irf3* mRNA levels in the liver, epididymal white adipose tissue and gastrocnemius muscle of CMI3KO mice compared to αMHC-Cre upon LAD determined by qPCR, Cre (n=5), CMI3KO (n=4), biological replicates.

b Relative *Irf3* mRNA levels in isolated cardiac fibroblasts, cardiac endothelial cells and other cells (supernatant pellet after cardiomyocytes and fibroblasts isolation) from CMI3KO and αMHC-Cre mice, n=4(fibroblasts), n=3(endothelial cells), n=3(other cells) .

c Body weight of male CMI3KO and αMHC-Cre mice upon LAD. Sham: n=7(αMHC-Cre), n=8(CMI3KO); LAD: n=7(αMHC-Cre), n=9(CMI3KO), biological replicates.

d Left ventricular posterior wall end systole analyzed by echocardiography in CMI3KO and αMHC-Cre mice upon LAD. Analyzed by one-way ANOVA with Šídák's multiple comparison test, n=8 (αMHC-Cre), n=8(CMI3KO). Cre-Sham vs. Cre-LAD: ****P*=0.0005; Cre-LAD vs. CMI3KO-LAD:**P*=0.0288.

e-f siRNA mediated knockdown of IRF3 in neonatal rat cardiomyocytes at mRNA (N=6 per group) and protein level (n=3 per group). mRNA knockdown: ****P*=6.6X10^{-5}; protein knockdown: ****P*=0006.

g Expression of IRF family members in neonatal rat cardiomyocytes upon siRNA mediated knockdown of *Irf3*, n=6 per group. *Irf3*: ****P*=0.00015, *Irf7*: ***P*=0.00311.

h Expression of *Irf3* target inflammatory genes in neonatal rat cardiomyocytes upon siRNA mediated knockdown of *Irf3* in the absence and presence LPS treatment, n=6 replicates per group. *P* values are shown next to the bar graph.

All data are represented as mean ± SEM. Statistical significance was assessed by unpaired two-tailed Student's t test (**e, f, g, h**). Source data file is provided.

Fig. S3: Muscle-specific activation of IRF3 results in impaired cardiac function.

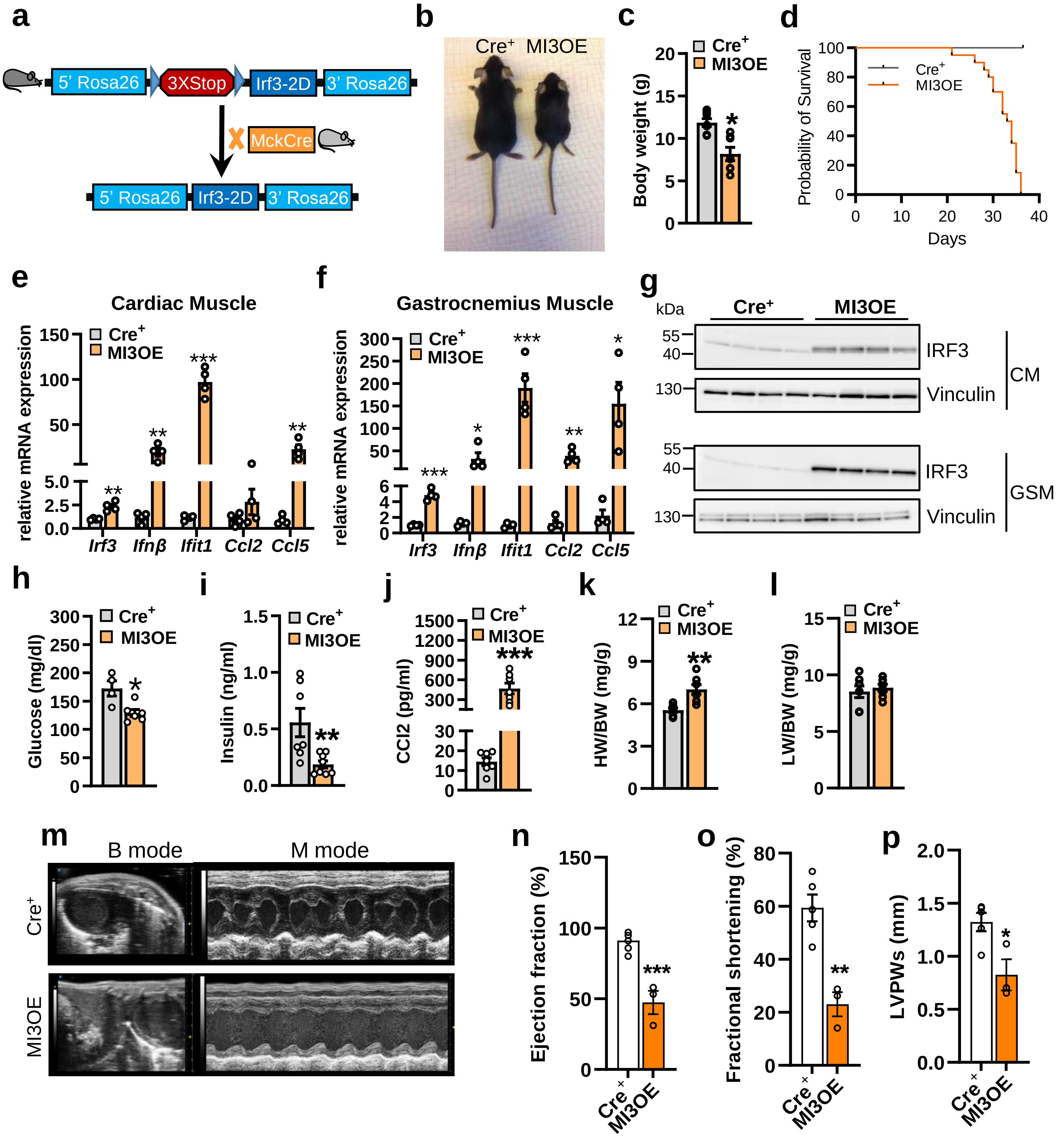

a Representative model showing generation strategy of MI3OE transgenic mouse.
b Gross morphology of control (Cre$^+$) and MI3OE mice at 4 weeks age.
c Body weight measured in control (Cre$^+$) and MI3OE mice, n=7 per group. ***P*=0.0017.
d Kaplan Meier plot showing survival curve of control (Cre$^+$) and MI3OE mice.
e Gene expression of *Irf3* and its target genes in left ventricular tissue determined by qPCR, n=4 per group. *Irf3*: ***P*=0.0020; *Ifnβ:* ***P*=0.0055; *Ifit1:* ****P*=1.9X10^{-5}; *Ccl5*: ***P*=0.0055.
f Gene expression of *Irf3* and its target genes in gastrocnemius tissue determined by qPCR, n=4 per group. *Irf3*: ****P*=.3.8X10^{-5}; *Ifnβ:* **P*=0.0582; *Ifit1:* ****P*=0.0010; *Ccl2*: ***P*=0.0025; *Ccl5:* **P*=0.0191.
g Immunoblot showing IRF3 protein levels in cardiac and gastrocnemius muscle, n=4 per group.
h-j Plasma glucose, insulin and CCL2 levels determined using glucometer, RIA and ELISA kit, respectively. Glucose: ***P*=0.0110; Insulin: ***P*=0.0096; CCL2: ****P*= 0.0001.
k-l Heart and lung weight to body weight ratio measured in the control (Cre$^+$) and MI3OE mice, n=7 per group. ***P*= 0.0019.

Fig. S3: Muscle-specific activation of IRF3 results in impaired cardiac function.

m Representative images from echocardiography performed in awake 4 week old control (Cre^+) and MI3OE mice.
n-p Cardiac function determined by echocardiography in awake 4 week old control (Cre^+) and MI3OE mice. Cre (n=5), MI3OE (n=3), biological replicates. %EF: ****P*=0.0011; %FS: ***P*=0.0028; %LVPWs: **P*=0.0194.

Survival curve, tissue weight and echocardiography experiments were performed using independent cohorts. All data are represented as mean ± SEM. Statistical significance was calculated by unpaired two-tailed Student's t test. Source data file is provided.

Fig. S4: Effect on inflammatory and mitochondrial oxidative pathways upon IRF3 activation in the cardiomyocytes of CMI3OE mice.

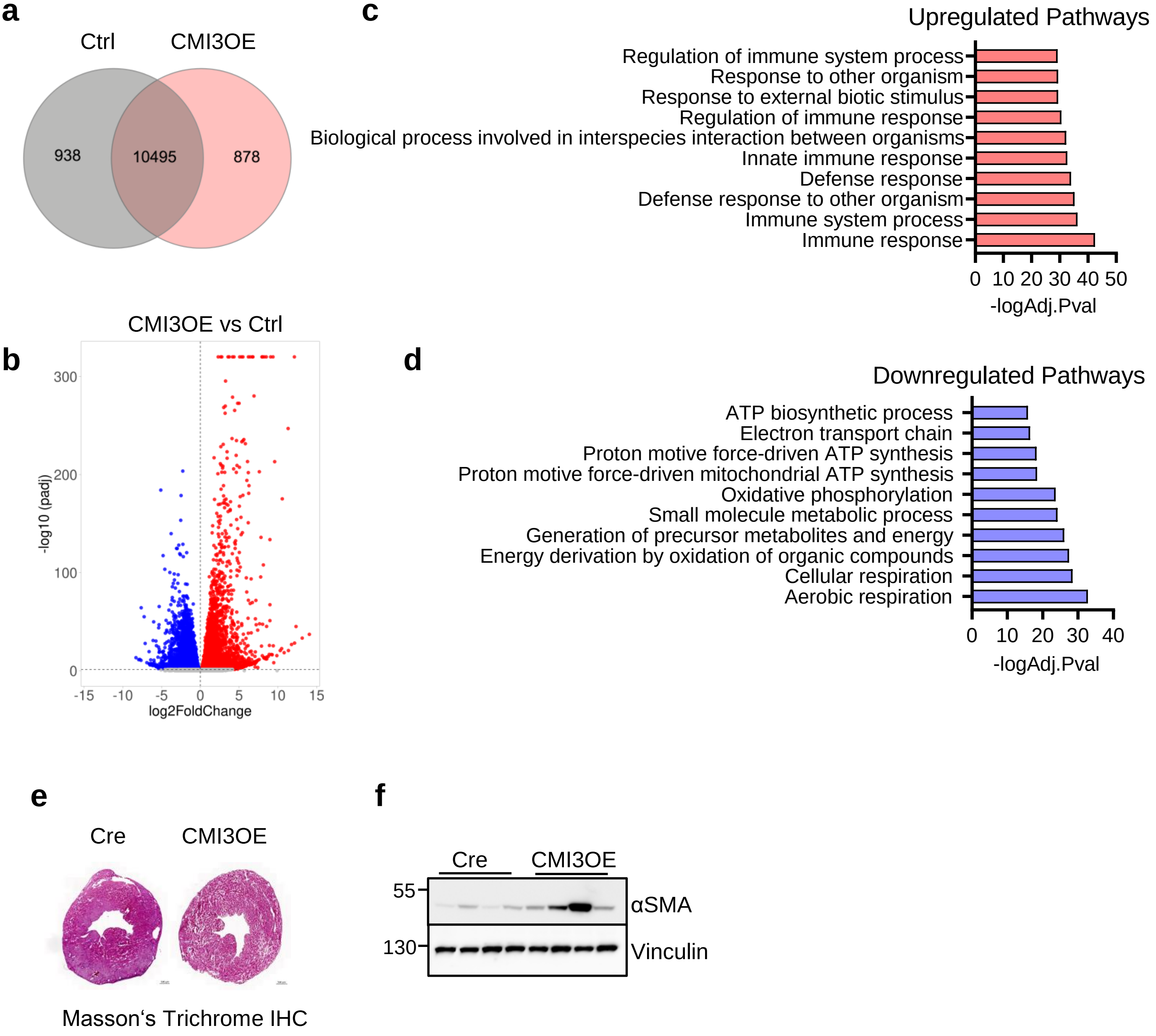

a Venn diagram showing number of differentially regulated genes in the left ventricle of CMI3OE compared to Cre control.
b Volcano plot showing differentially regulated genes in the left ventricle of CMI3OE compared to Cre control.
c GO pathway analysis using differentially upregulated genes in the left ventricle of CMI3OE.
d GO pathway analysis using differentially downregulated genes in the left ventricle of CMI3OE.
e Masson’s trichrome IHC in CMI3OE cardiac tissue compared to Cre control.
f Immunoblot showing expression of αSMA in CMI3OE compared to Cre control in the left ventricular tissue. N=4 per group, biological replicates.

Fig. S5: Effect on inflammation and mitochondrial marker genes upon IRF3-2D expression in cardiomyocytes.

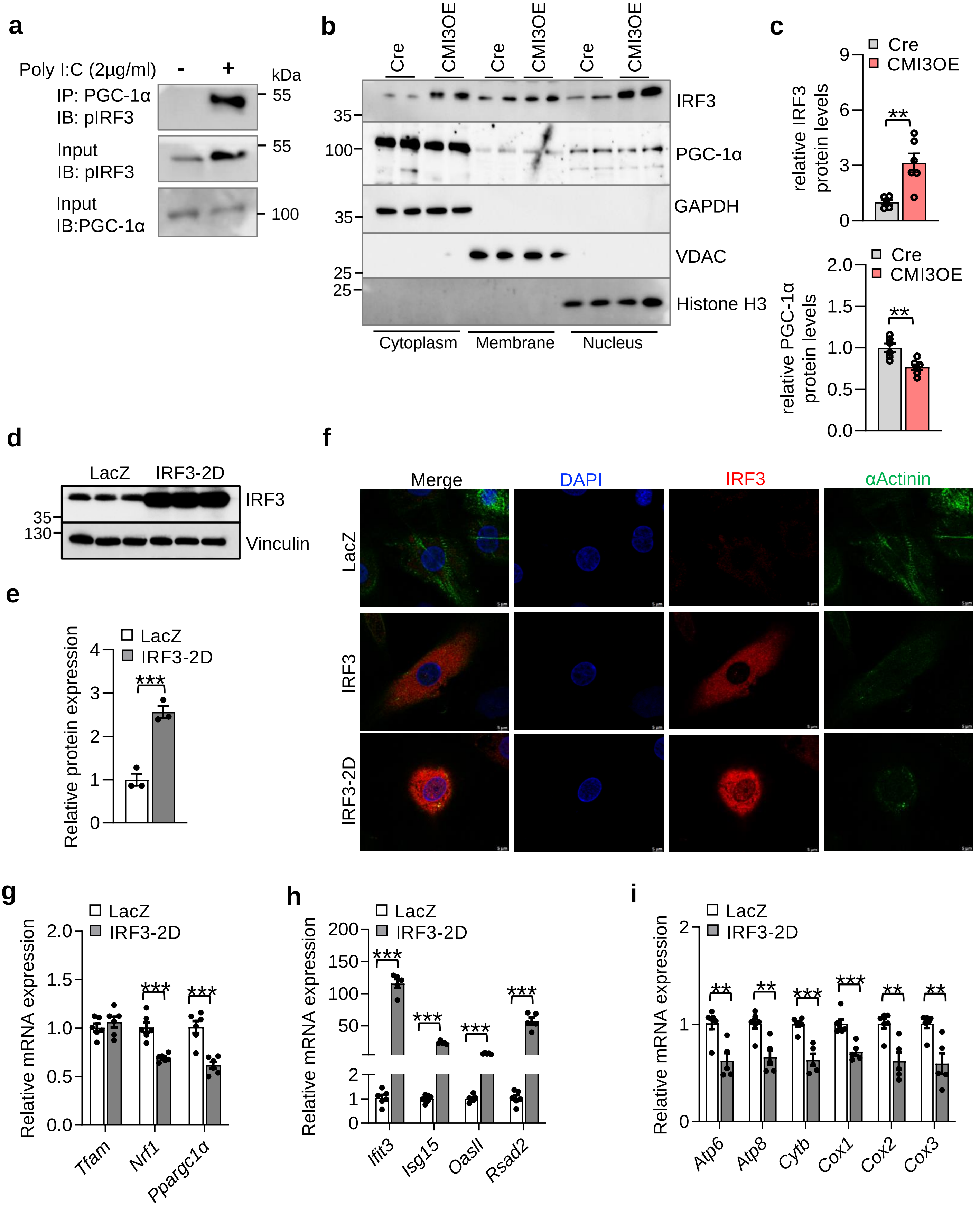

Fig. S5: Effect on inflammation and mitochondrial marker genes upon IRF3-2D expression in cardiomyocytes.

a Immunoblot showing co-immunoprecipitation assay in NRCMs stimulated with Poly I:C (2µg/ml) for 4h. Immunoprecipitation was performed with anti-PGC-1α antibody.

Fig. S5: Effect on inflammation and mitochondrial marker genes upon IRF3-2D expression in cardiomyocytes.

b Immunoblot showing cell fractionation and expression of IRF3 in the cytoplasm, membrane and nuclear fraction of adult cardiomyocytes isolated from CMI3OE mice compared to Cre control. GAPDH, VDAC and Histone H3 were used to detect cytoplasmic, membrane and nuclear purity. n=2 per group, biological replicates.

c Quantification of IRF3 and PGC-1α pooled protein levels in the immunoblot shown in Fig. S5b from the cytoplasm, membrane, and nuclear fractions of cardiomyocytes isolated from CMI3OE mice. IRF3: **P=0.0025; PGC-1α: **P=0.0039

d Immunoblot showing expression of IRF3-2D in neonatal rat cardiomyocytes (NRCMs).

e Quantification of the immunoblot shown in Fig. S5d. N=3 per group. ***P=0.0014.

f Immunofluorescence images showing cellular localization of IRF3 upon expression of wild-type IRF3 or IRF3-2D in NRCMs compared to LacZ control.

g Expression of mitochondrial biogenesis marker genes in NRCMs upon IRF3-2D expression. n=6 per group. *Nrf1*: ***P=0.0002; *Ppargc1α*: ***P=0.0003.

h Expression of IRF3 target genes in NRCMs by qPCR upon IRF3-2D expression. LacZ (n=6), IRF3-2D (n=5). *Ifit3*: ***$P=1.9\times10^{-8}$; *Isg15*: ***$P=2.8\times10^{-9}$; *OasII*: ***$P=3.5\times10^{-8}$; *Rsad2*: ***$P=1.5\times10^{-6}$.

i Gene expression of mitochondrial marker genes in NRCMs upon IRF3-2D expression. LacZ (n=6), IRF3-2D (n=5). *Atp6*: **P=0.0036; *Atp8*: **P=0.0032; *Cytb*: ***P=0.0003; *Cox1:* ***P=0.0010; *Cox2*: **P=0.0031; *Cox3*: **P=0.0044.

All data are represented as mean ± SEM. Statistical significance was calculated by unpaired two-tailed Student’s t test. Source data file is provided.

Fig. S6: Effect on inflammation and mitochondrial marker genes upon PGC-1α expression in cardiomyocytes.

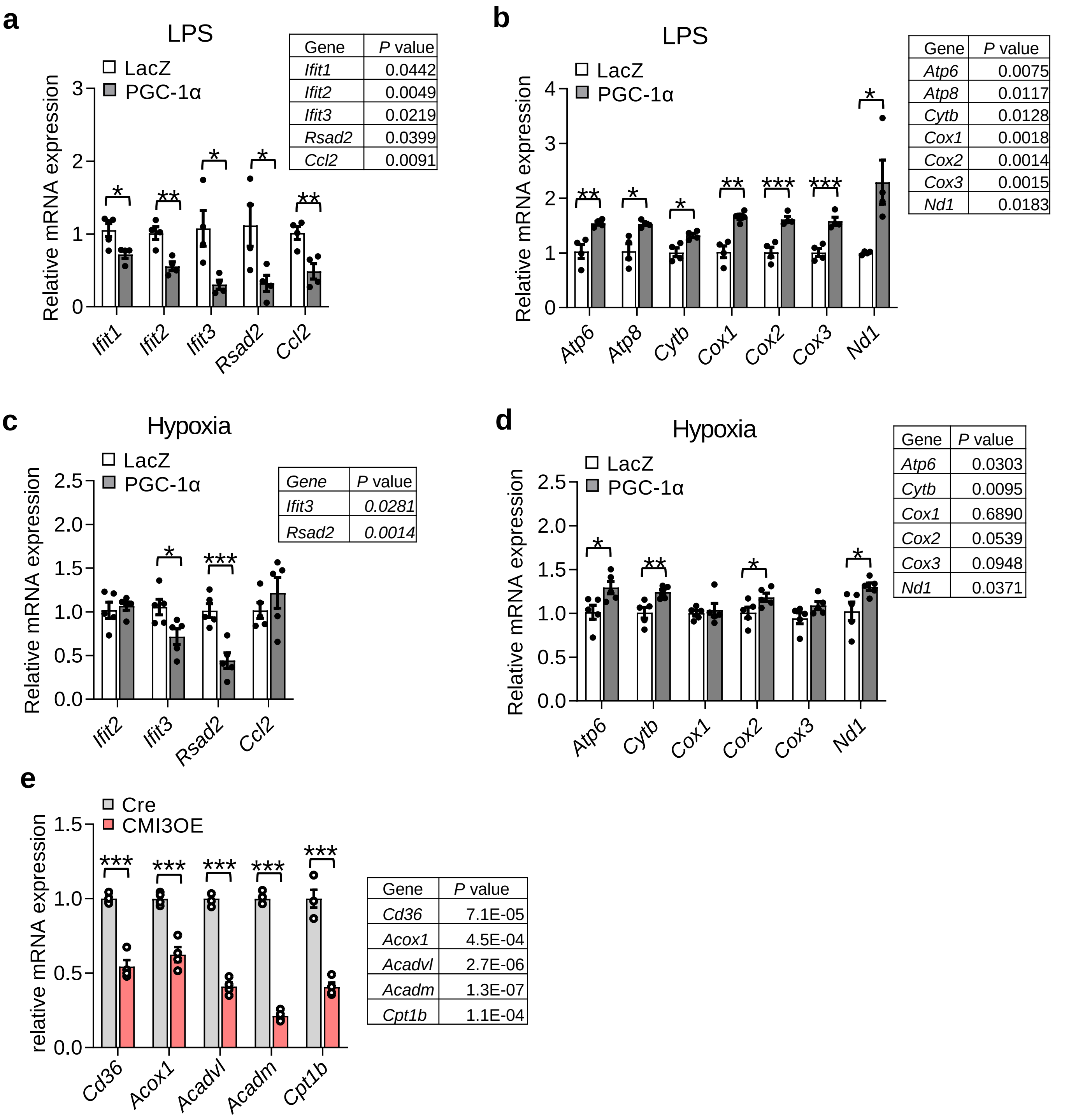

a

Gene	*P* value
Ifit1	0.0442
Ifit2	0.0049
Ifit3	0.0219
Rsad2	0.0399
Ccl2	0.0091

b

Gene	*P* value
Atp6	0.0075
Atp8	0.0117
Cytb	0.0128
Cox1	0.0018
Cox2	0.0014
Cox3	0.0015
Nd1	0.0183

c

Gene	*P value*
Ifit3	*0.0281*
Rsad2	*0.0014*

d

Gene	*P* value
Atp6	0.0303
Cytb	0.0095
Cox1	0.6890
Cox2	0.0539
Cox3	0.0948
Nd1	0.0371

e

Gene	*P* value
Cd36	7.1E-05
Acox1	4.5E-04
Acadvl	2.7E-06
Acadm	1.3E-07
Cpt1b	1.1E-04

a Expression of IRF3 target genes by qPCR in LPS treated NRCMs upon PGC-1α expression. n=4 per group. *P* values are shown next to the bar graph.
b Expression of mitochondrial oxidative phosphorylation marker genes by qPCR in LPS treated NRCMs upon PGC-1α expression. n=4 per group. *P* values are shown next to the bar graph.
c Expression of IRF3 target genes by qPCR in NRCMs expressing PGC-1α and subjected to hypoxia. N=5 per group. *P* values are shown next to the bar graph.
d Expression of mitochondrial oxidative phosphorylation marker genes by qPCR in NRCMs expressing PGC-1α and subjected to hypoxia. n=5 per group. *P* values are shown next to the bar graph.
e Expression of PPARα target genes in the left ventricle of CMI3OE mice. n=4 per group. *P* values are shown next to the bar graph.
All data are represented as mean ± SEM. Statistical significance was calculated by unpaired two-tailed Student's t test. Source data file is provided.

Fig. S7: Cardiac metabolic alterations and adaptions upon IRF3 activation in cardiomyocytes.

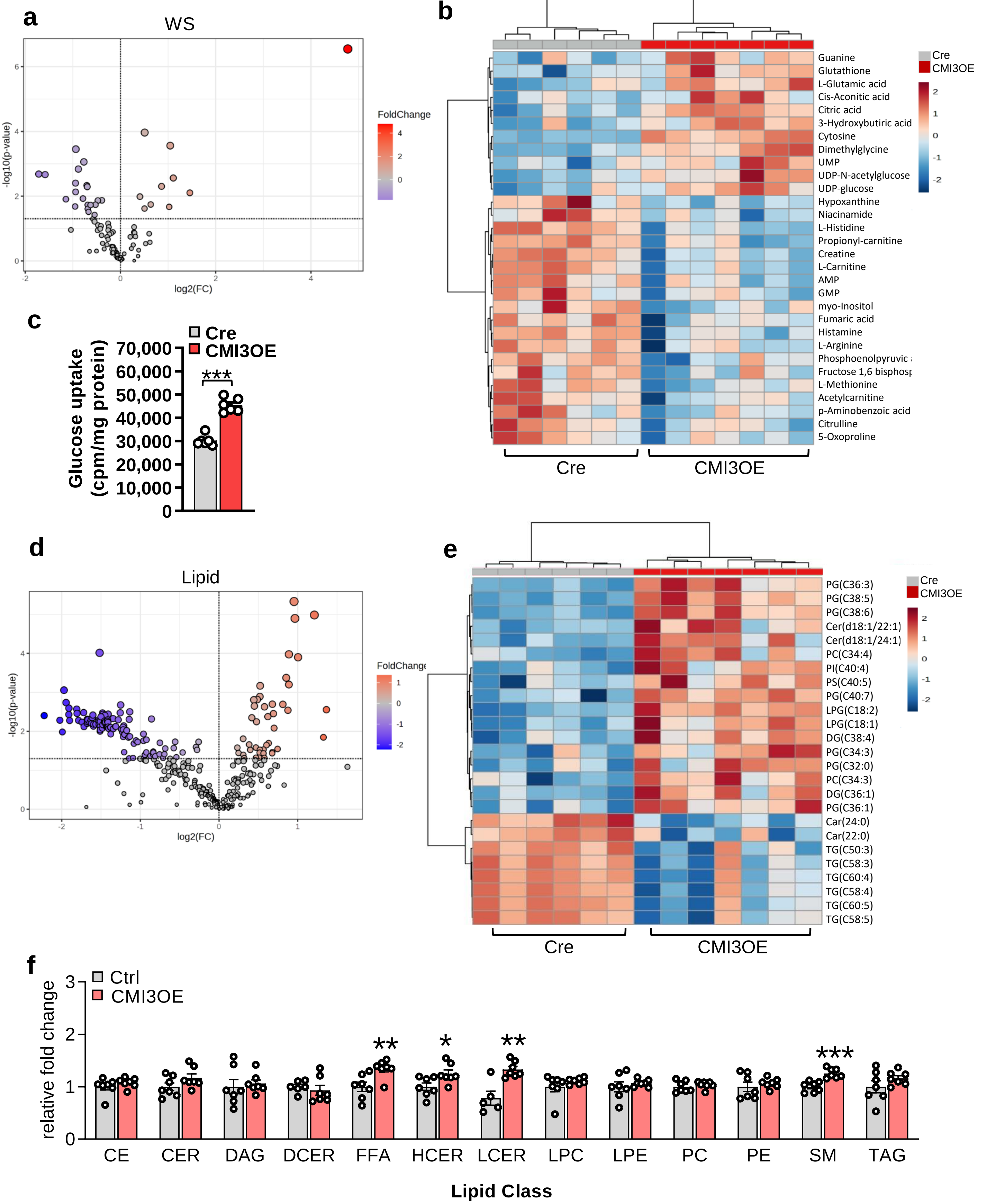

Fig. S7: Cardiac metabolic alterations and adaptions upon IRF3 activation in cardiomyocytes.
a Volcano plot showing the water soluble metabolites in CMI3OE compared to αMHCMCM mice cardiac tissue.
b Heatmap showing top 30 water soluble metabolites altered in CMI3OE compared to αMHCMCM mice cardiac tissue. Cre (n=6), CMI3OE (n=7).

Fig. S7: Cardiac metabolic alterations and adaptions upon IRF3 activation in cardiomyocytes.
c Glucose uptake using ^{3}H-deoxyglucose in adult cardiomyocytes isolated from 12 wk old CMI3OE and Cre control mice. Cardiomyocytes were isolated from n=3 per group and each mouse cardiomyocytes were seeded in two 12 well plates. N=6wells/group. ***$P=2X10^{-6.}$
d Volcano plot showing lipid metabolites in CMI3OE compared to αMHCMCM mice cardiac tissue.
e Heatmap showing top 25 lipid metabolites altered in CMI3OE compared to αMHCMCM mice cardiac tissue. Cre (n=6), CMI3OE (n=7).
f Lipidomic analysis showing relative fold change in plasma lipid class concentration analyzed on Lipidyzer in 12 wk old male CMI3OE mice compared to control mice. Ctrl (n=7), CMI3OE (n=7) are biological replicates. FFA: ***P*=0.0082; HCER: **P*=0.0279; LCER: ***P*=0.0019; SM: ****P*=0.0003.
All data are represented as mean ± SEM. Statistical significance was calculated by unpaired two-tailed Student's t test. Source data file is provided.

Fig. S8: Gene expression alterations in the left ventricle of CMI3OE mice.

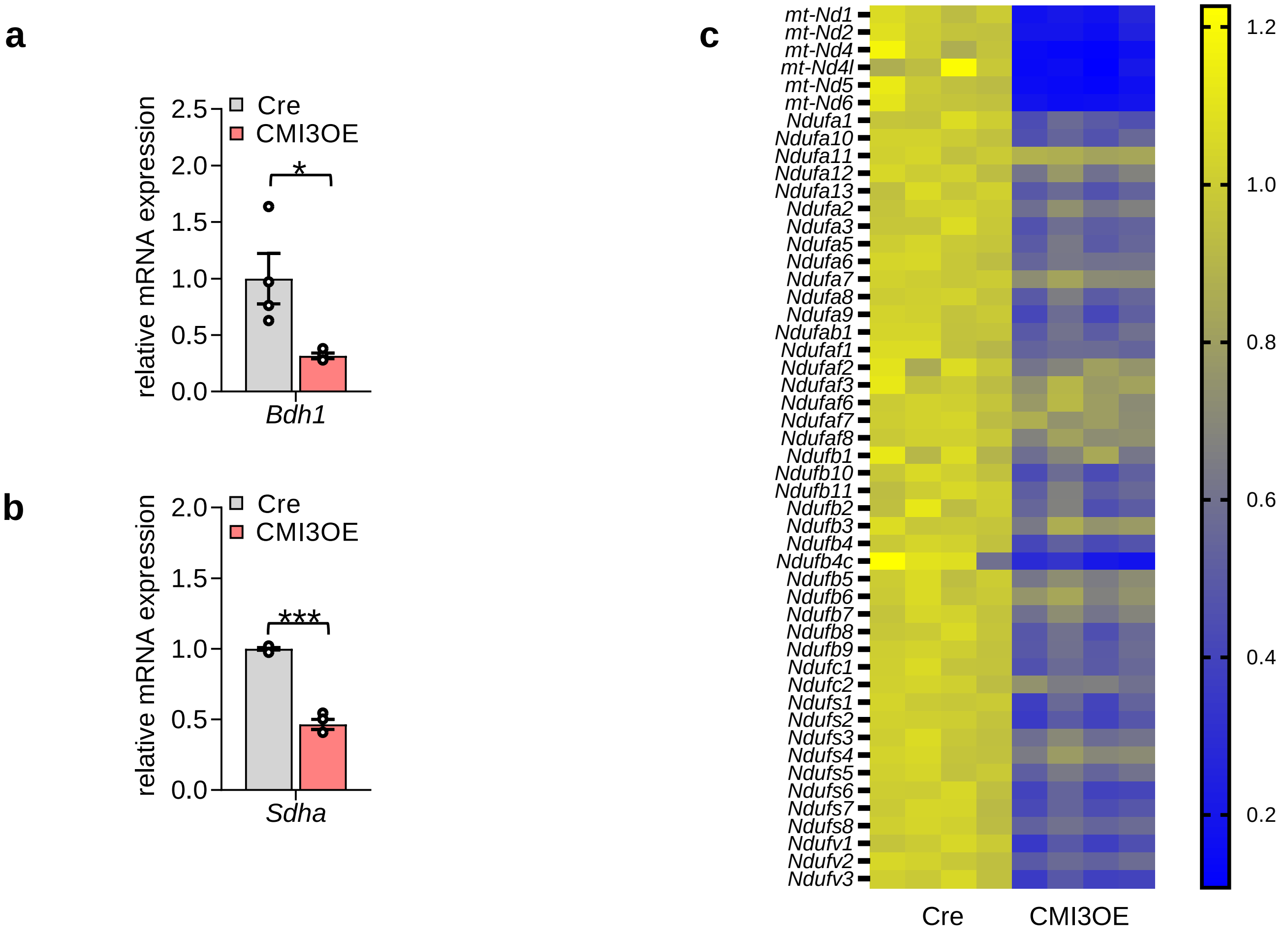

a Gene expression of *Bdh1* in the left ventricle of CMI3OE mice compared to αMHCMCM. N=4 per group. *P=0.0227.

b Gene expression of *Sdha* in the left ventricle of CMI3OE mice compared to αMHCMCM. N=4 per group. ***P=5.9X10^{-6}.

c Heatmap showing differential expression of NAD dehydrogenase complex assembly marker genes in CMI3OE mice compared to αMHCMCM. N=4 per group.

All data are represented as mean ± SEM. Statistical significance was calculated by unpaired two-tailed Student's t test. Source data file is provided.

Fig. S9: Stable isotope in cardiomyocytes isolated from adult CMI3OE mice.

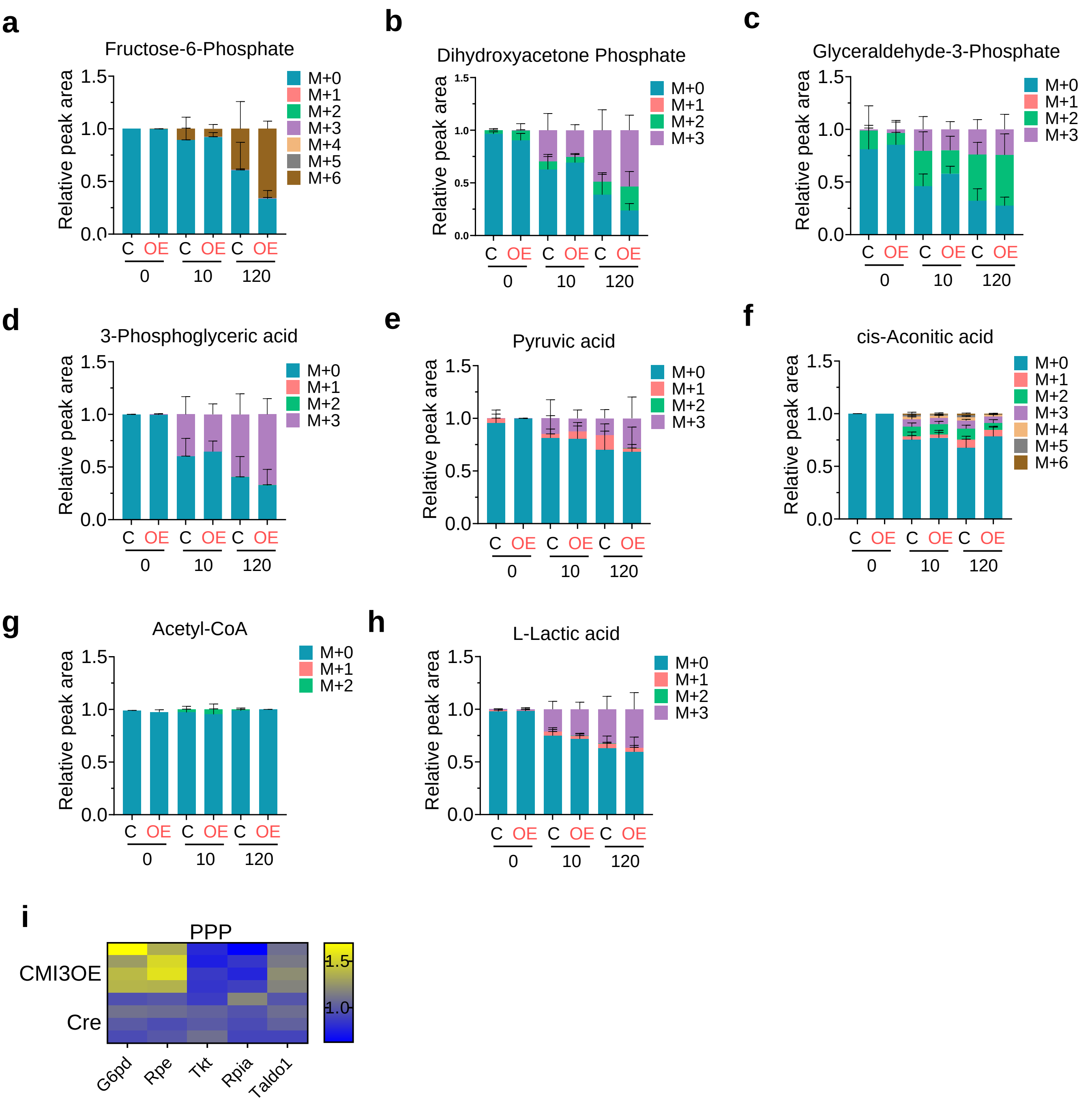

a-h Metabolic flux assay in adult cardiomyocytes isolated from Cre control and CMI3OE mice using U-$^{13}C_6$-Glucose at 10 min for glycolysis pathway and 120min for TCA cycle. Samples group, 0min: Cre (n=4), CMI3OE (n=4); 10min: Cre (n=5), CMI3OE (n=6); 120min: Cre (n=5), CMI3OE (n=4), independent biological replicates. .

i Gene expression analysis showing effect on PPP marker genes mRNA level in the left ventricle of CMI3OE mice compared to Cre control. n=4 per group.

Fig. S10: Cardiomyocyte-specific moderate PGC-1α expression improves cardiac function in adult CMI3OE mice.

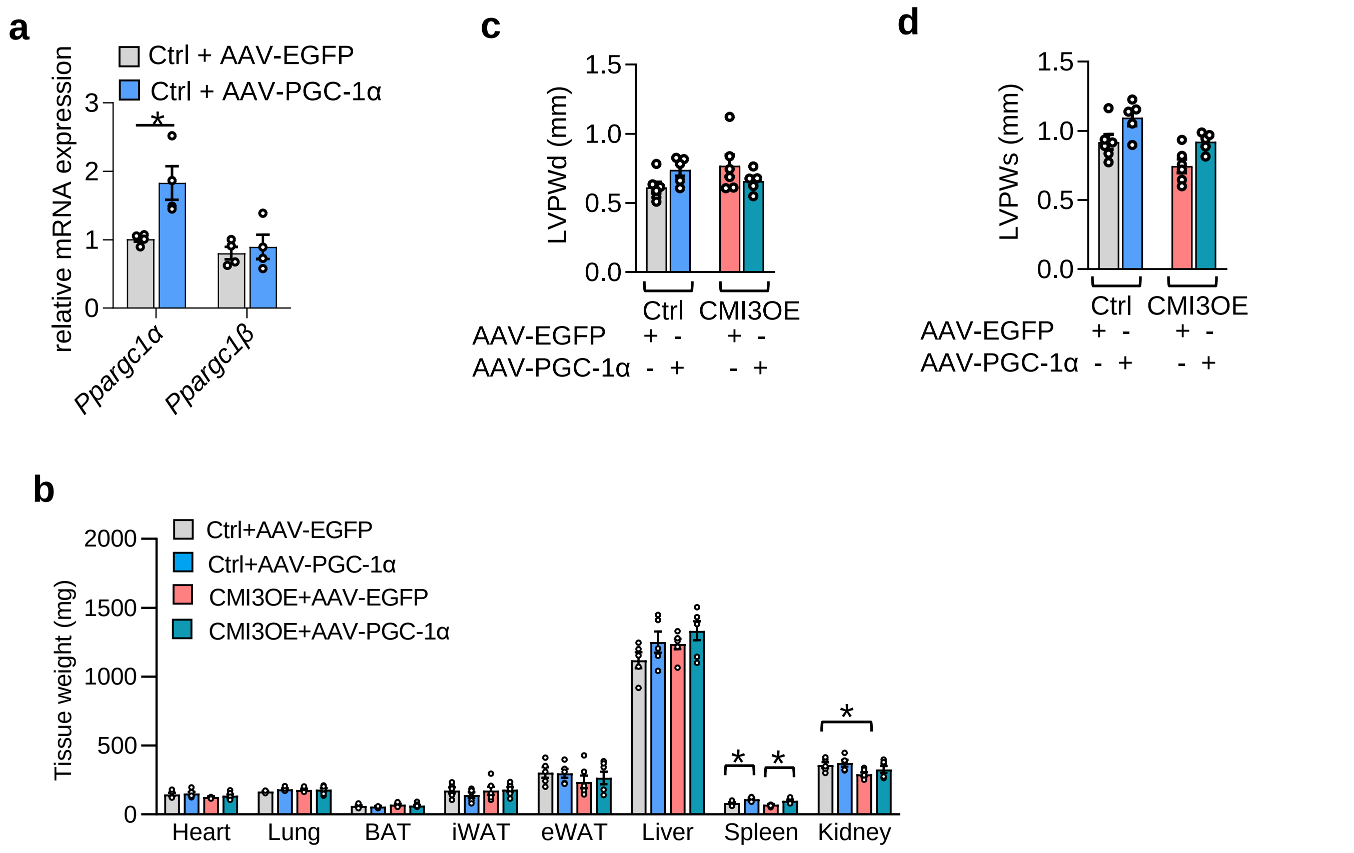

a Gene expression of *Ppargc1α* and *Ppargc1β* in the left ventricular tissue of control mice using AAV9-TnT-PGC-1α compared to AAV9-TnT-EGFP determined by qPCR analysis. N=4 per group. *P=0.0170.

b Tissue weight of CMI3OE treated with AAV9-TnT-PGC-1α compared to AAV9-TnT-EGFP controls. n=5 (Ctrl-AAV-EGFP), n=5 (Ctrl-AAV-PGC-1α), n=6 (CMI3OE-AAV-EGFP), n=5 (CMI3OE-AAV-PGC-1α) . Spleen: Ctrl-AAV-EGFP vs Ctrl-AAV-PGC-1α, *P=0256; CMI3OE-AAV-EGFP vs CMI3OE-AAV-PGC-1α, *P=0256; Kidney: Ctrl-AAV-EGFP vs CMI3OE-AAV-EGFP, *P=0230.

c-d Left ventricular posterior wall end diastole (LVPWd) and left ventricular posterior wall end systole (LVPWs) measurement in CMI3OE treated with AAV9-TnT-PGC-1α compared to AAV9-TnT-EGFP controls. n=6 (Ctrl-AAV-EGFP), n=5 (Ctrl-AAV-PGC-1α), n=6 (CMI3OE-AAV-EGFP), n=5 (CMI3OE-AAV-PGC-1α), biological replicates.

Fig. S11: PGC-1a expression attenuates cardiac inflammation and upregulates mitochondrial OXPHOS levels in CMI3OE mice.

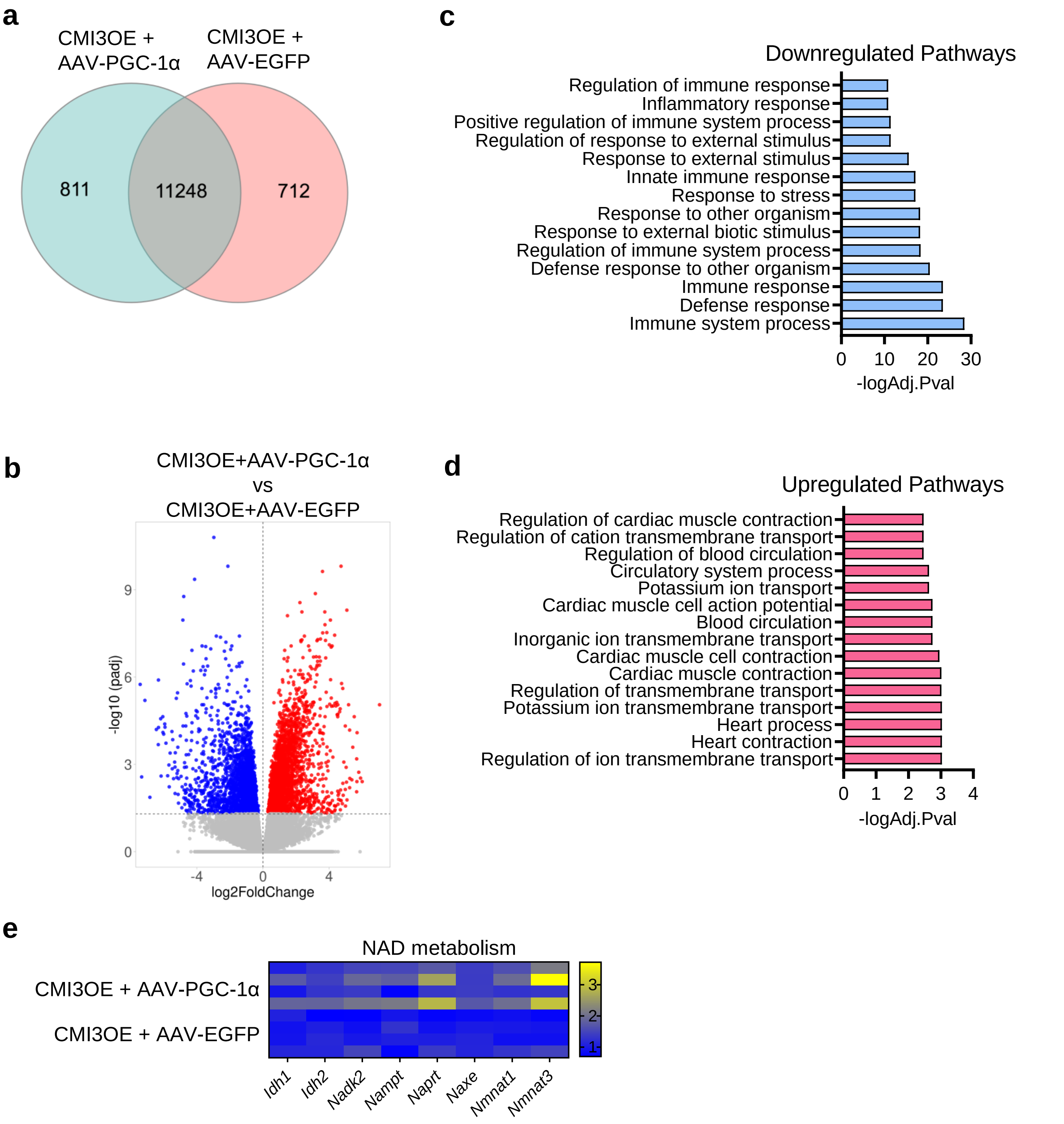

a Venn diagram showing number of differentially regulated genes in the left ventricle of CMI3OE-AAV-PGC-1α compared to CMI3OE-AAV-EGFP mice.
b Volcano plot showing differentially regulated genes in the left ventricle of CMI3OE-AAV-PGC-1α compared to CMI3OE-AAV-EGFP mice.
c GO pathway analysis using differentially downregulated genes in the left ventricle of CMI3OE-AAV-PGC-1α compared to CMI3OE-AAV-EGFP mice.
d GO pathway analysis using differentially upregulated genes in the left ventricle of CMI3OE-AA-V-PGC-1α compared to CMI3OE-AAV-EGFP mice.
e Differential expression of genes regulating NAD metabolism in the left ventricle of CMI3OE-AAV-PGC-1α compared to CMI3OE-AAV-EGFP mice.

Created by https://lipidomicstandards.org, version v2.5.0

Contents of Report

Separation Workflow 1
Overall study design . . . 1
Lipid extraction . . . 1
Analytical platform . . . 1
Quality control . . . 2
Method qualification and validation . . . 2
Reporting . . . 2

Sample Descriptions 2
CMI3OE and aMHCMCM Control left ventricle tissue / Mouse / Tissues (e.g., liver, heart, brain) . . . 2

Separation Workflow

Overall study design

Title of the study	IRF3 activation in cardiomyocytes impairs mitochondrial oxidative function through PGC-1α inhibition and drives heart failure		
Document creation date	12/11/2025	Principal investigator	Manju Kumari and Almut Schulze
Institution	Department of Cardiology, Angiology and Pneumology, University Hospital Heidelberg, Germany; German Cancer Research Center, Heidelberg, Germany; Department of Biochemistry and Molecular Cell Biology, University Medical Center Hamburg-Eppendorf, Germany	Corresponding Email	manju.kumari@med.uni-heidelberg.de
Is the workflow targeted or untargeted?	Targeted	Clinical	No

Lipid extraction

Extraction method	2-phase system	pH adjustment	Hydrochloric acid
2-phase system	Bligh&Dyer	Special conditions	Standard extraction conditions
Derivatization	None	Were internal standards added prior extraction?	Yes

Analytical platform

Ionizaton additives	Ammonium acetate	Number of separation dimensions	One dimension
Separation type 1	LC	Separation mode 1 (liquid)	HILIC for water soluble metabolites and RP for lipids
Detector	Mass spectrometer	MS type	Orbitrap
MS vendor	Thermo	Ion source	ESI
MS Level	MS^2	Mass window for precursor ion isolation (in Da total isolation window)	1.5
Mass resolution for detected ion at MS^2	High resolution	Resolution at m/z 200 at MS^2	17500
Mass accuracy in ppm at MS^2	5	Recording mode of raw data at MS^2	Profile mode
Was/Were additional dimension/techniques used	No		

Quality control

Blanks	Yes	Type of Blanks	Extraction blank
Quality control	Yes	Type of QC sample	Sample pool

Method qualification and validation

Method validation	No

Reporting

Are reported raw data uploaded into repository?	No	Are metadata available?	Available on request
Raw data upload	Yes		

Sample Descriptions

CMI3OE and aMHCMCM Control left ventricle tissue / Mouse / Tissues (e.g., liver, heart, brain)

Tissue type	left ventricle tissue	Storage and collection conditions	Available
Sample homogenization	No	Provided preanalytical information	Time to freeze, Preservation method
Temperature handling original sample	Room temperature	Instant sample preparation	No
Time to freeze	between 4 and 5 min	Snap freezing in liquid N2	Yes
Storage temperature	-80 °C	Additives	None
