# Supplementary material for: Activation of IRF3 in cardiomyocytes impairs mitochondrial oxidative function through PGC-1α inhibition and drives heart failure
Source: Nat Commun. 2026 Feb 27;17:2051. doi: 10.1038/s41467-026-69792-4 (PMC12948977; doi:10.1038/s41467-026-69792-4)
Supplement: Supplementary file 2 — Description Of Additional Supplementary File [file 41467_2026_69792_MOESM2_ESM.pdf]

Description of Additional supplementary files

Supplementary data 1:

Supplemental Table_primer list_excel

Supplementary data 2:

Unedited Immunoblots
