# Supplementary material for: Activation of IRF3 in cardiomyocytes impairs mitochondrial oxidative function through PGC-1α inhibition and drives heart failure
Source: Nat Commun. 2026 Feb 27;17:2051. doi: 10.1038/s41467-026-69792-4 (PMC12948977; doi:10.1038/s41467-026-69792-4)

**Uncut immunoblots used in this manuscript.**

**Fig 1a**

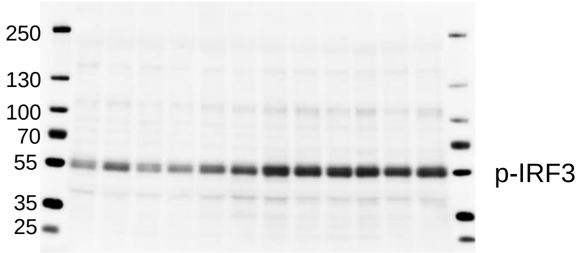


**Fig 1a**

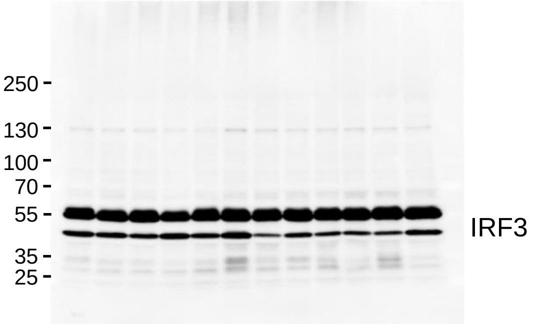


**Fig 1a**

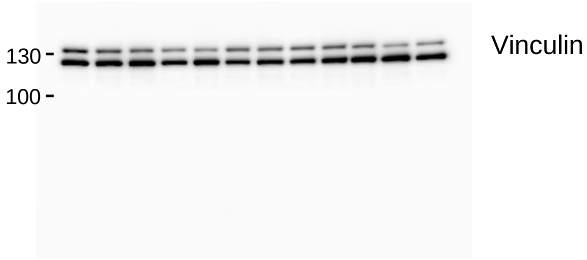


**Fig 1f**

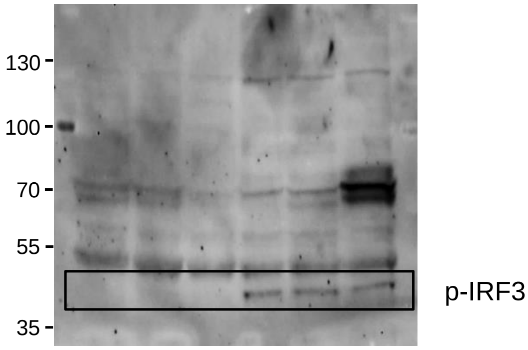


**Fig 1f**

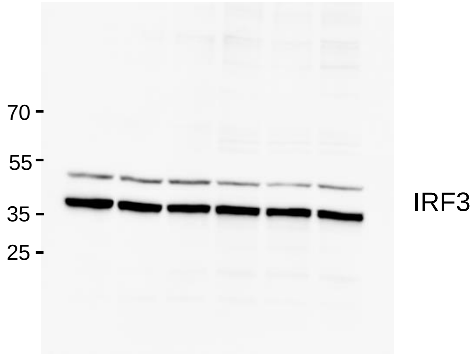


**Fig 1f**

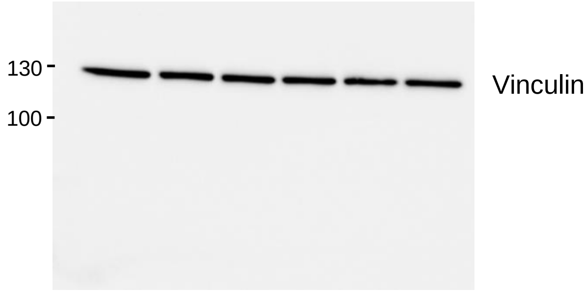


**Fig 1h**

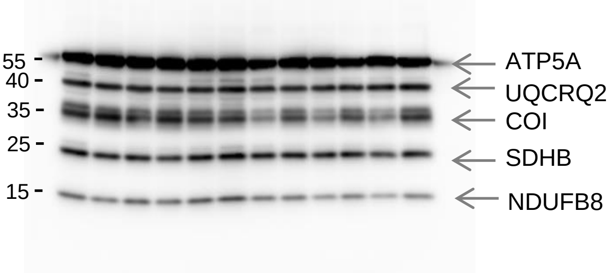


**Fig 1h**

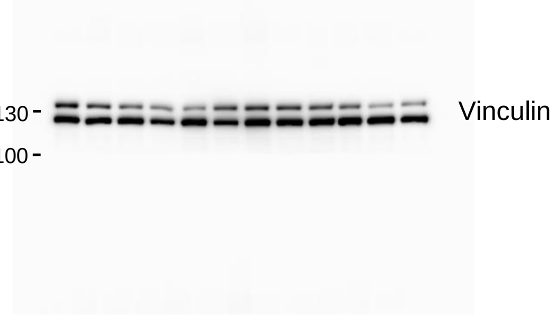


**Fig1l**

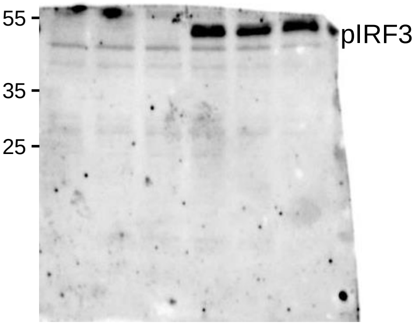


**Fig1l**

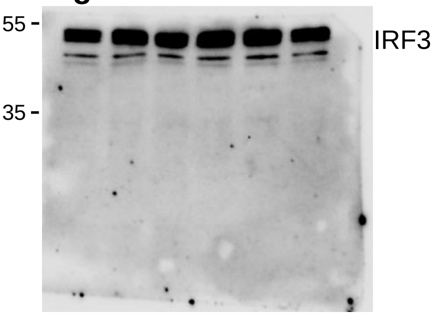


**Fig1l**

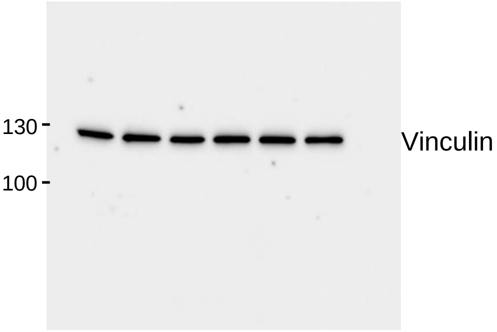

**Fig 2c**

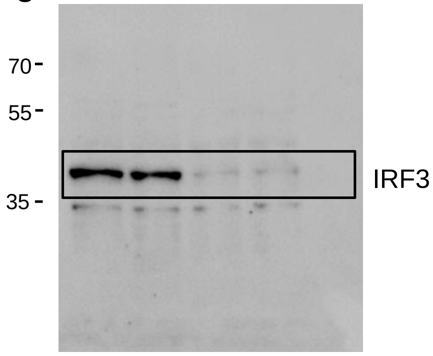


**Fig 2c**

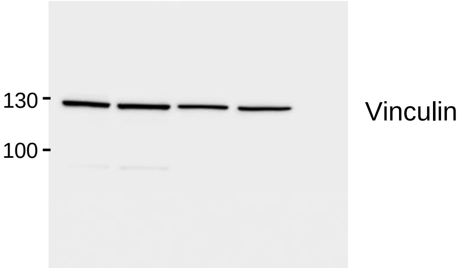


**Fig 2c**

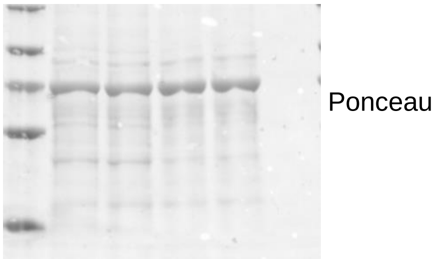


**Fig 3e**

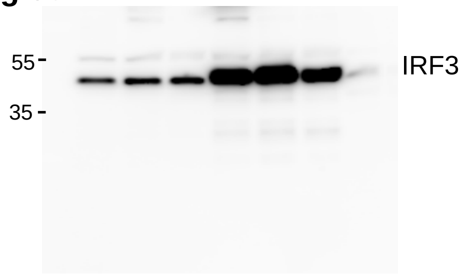


**Fig 3e**

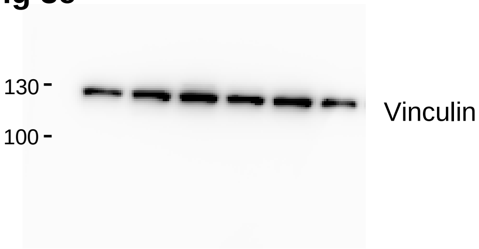


**Fig 4c**

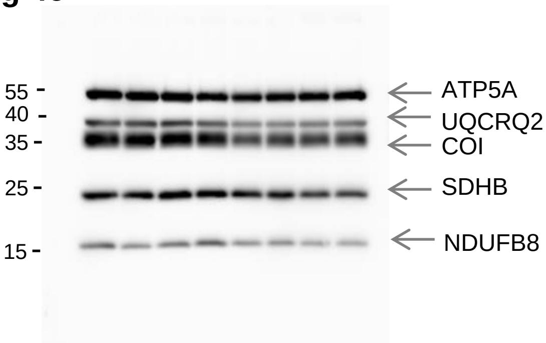


**Fig 4c**

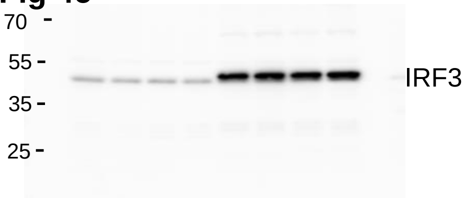


**Fig 4c**

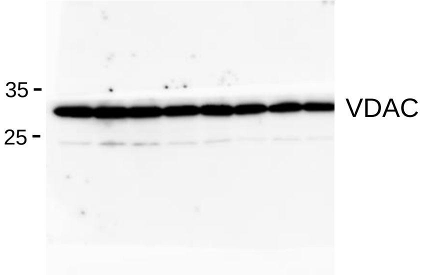


**Fig 4i**

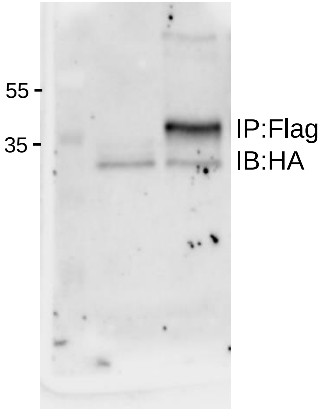


**Fig 4i**

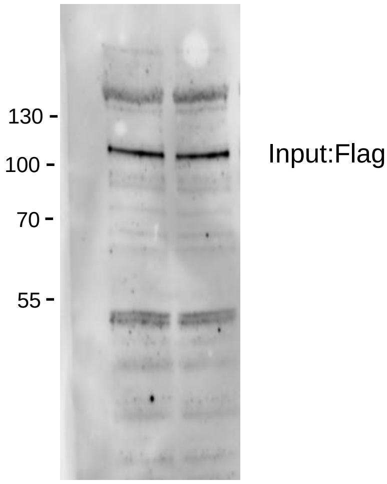


**Fig 4i**

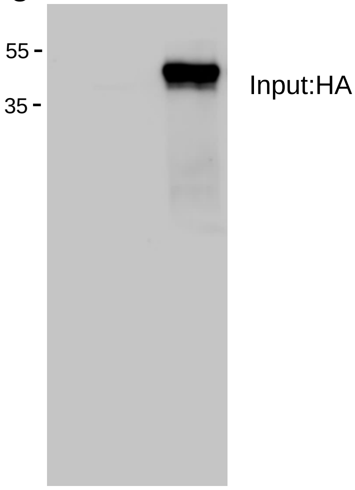

Fig 4j

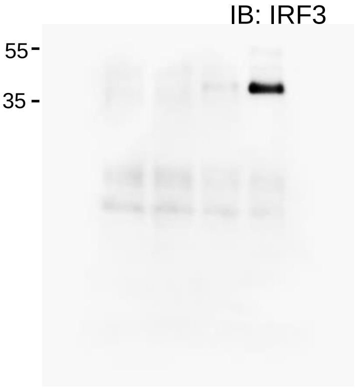


Fig 4j

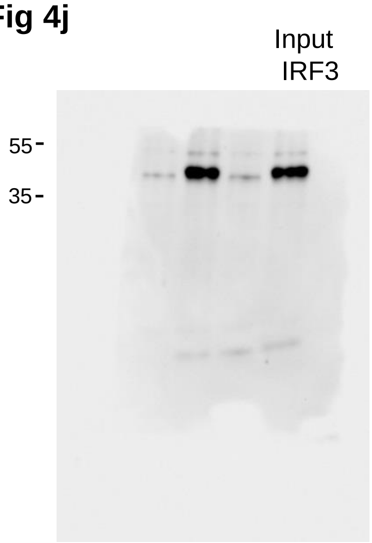


Fig 4j

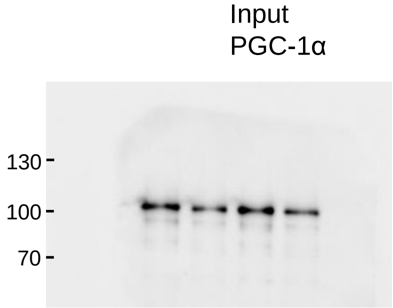


Fig 5i

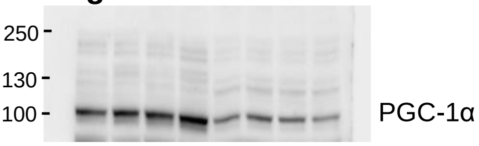


Fig 5i

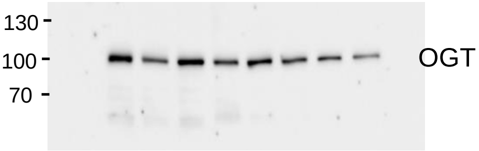


Fig 5i

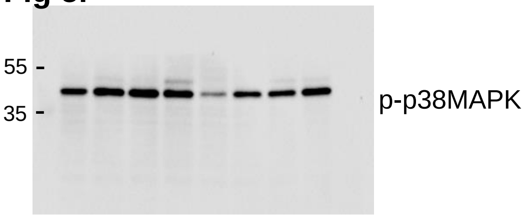


Fig 5i

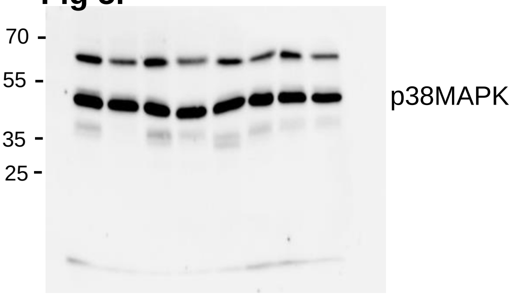


Fig 5i

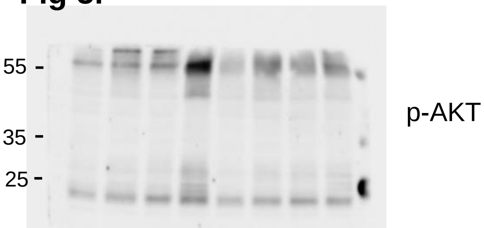


Fig 5i

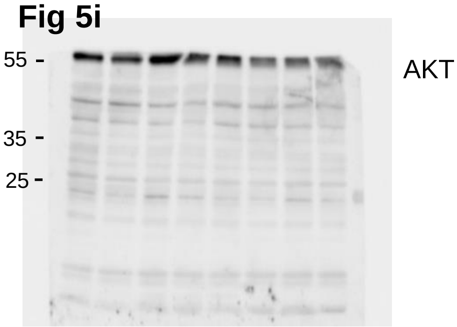


Fig 5i

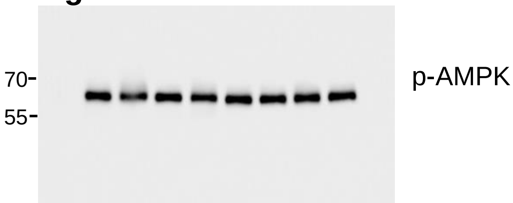


Fig 5i

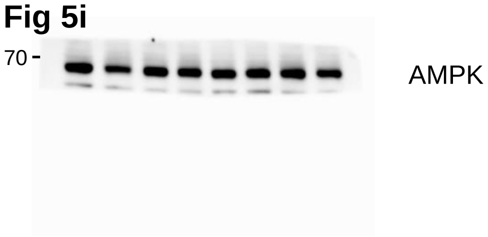


Fig 5i

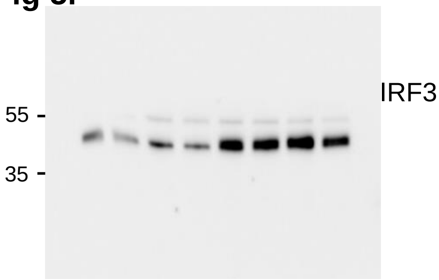


Fig 5i

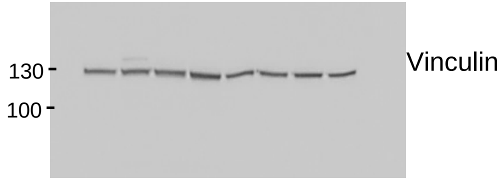


Fig 5i

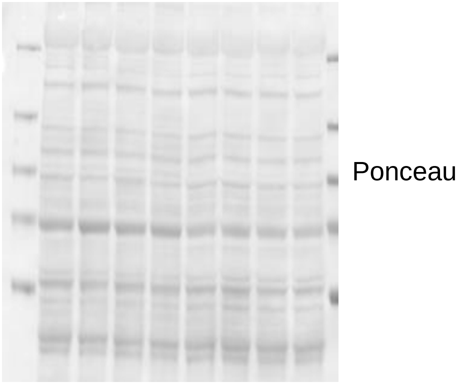

**Fig 5j**

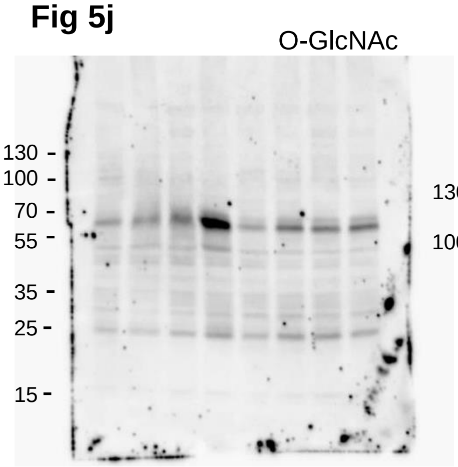


**Fig 5j**

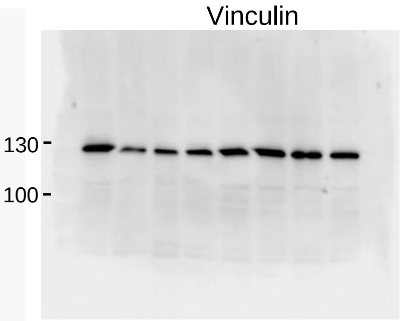


**Fig 5j**

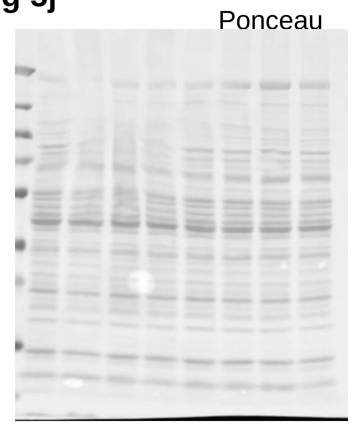


**Fig 9d**

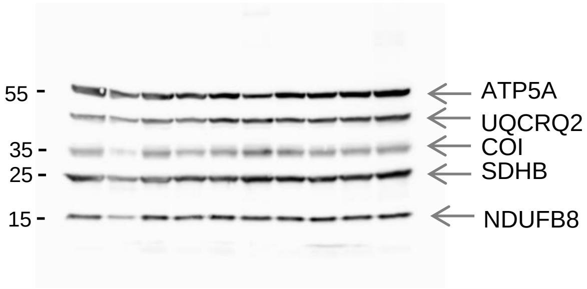


**Fig 9d**

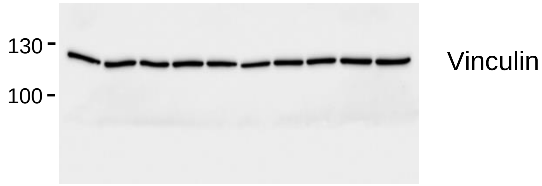


**Fig 9d**

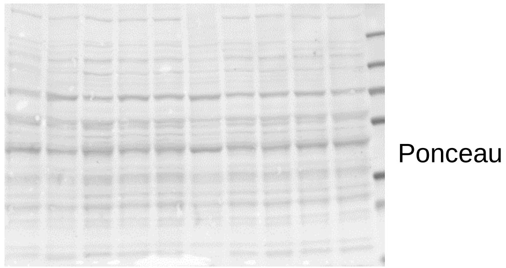

## Fig S1c

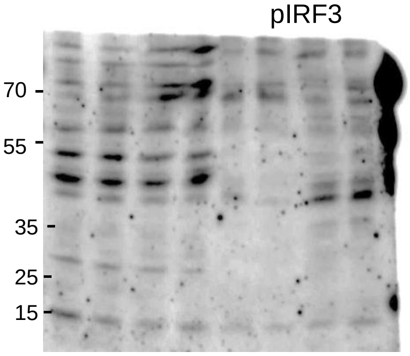


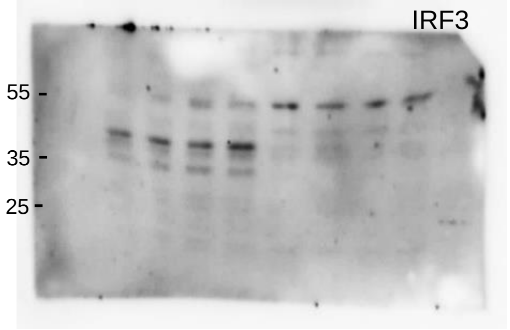


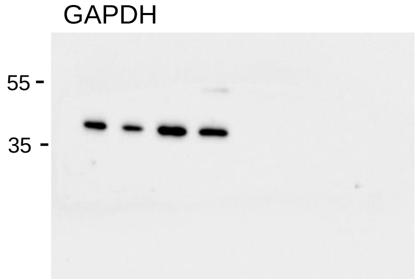


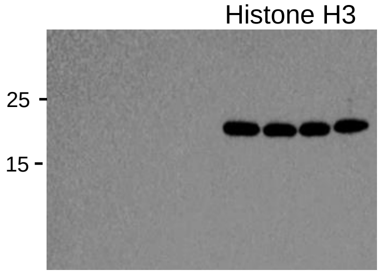


## Fig S2f

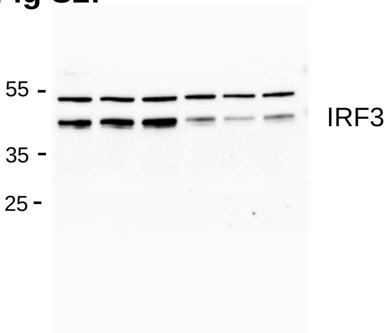


## Fig S3g

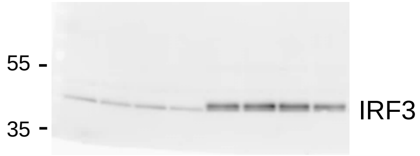


## Fig S3g

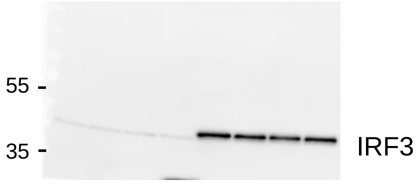


## Fig S2f

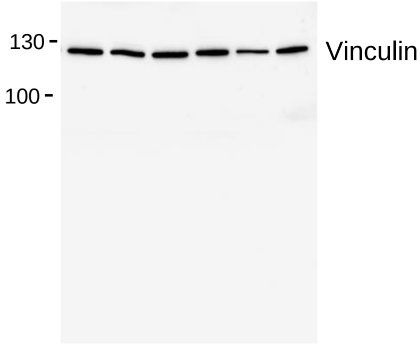


## Fig S3g

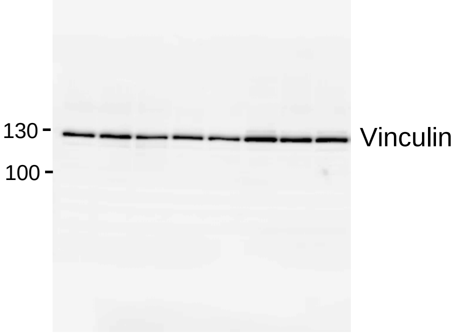


## Fig S3g

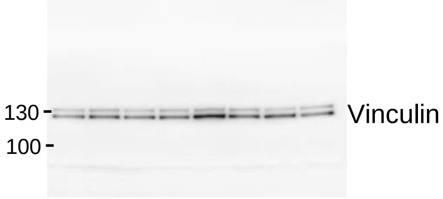


## Fig S4f

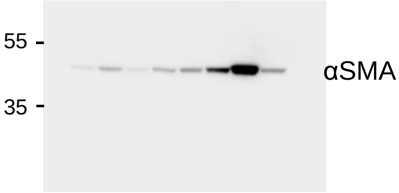


## Fig S4f

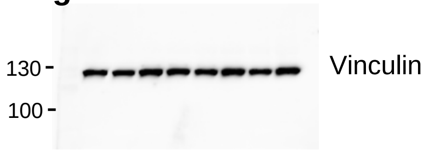

## Fig S5a

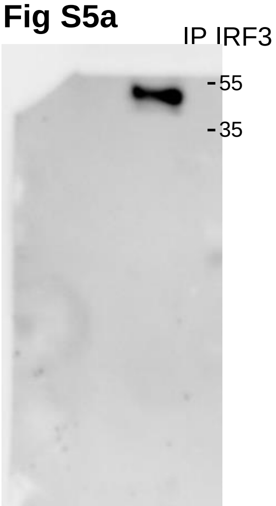


## Fig S5a

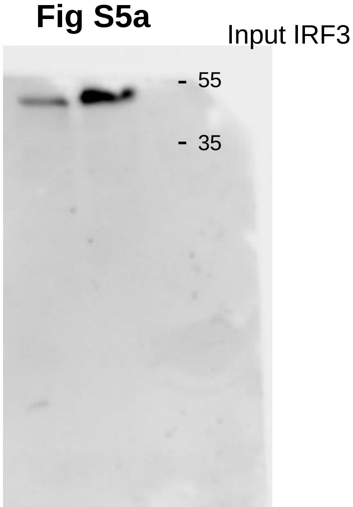


## Fig S5a

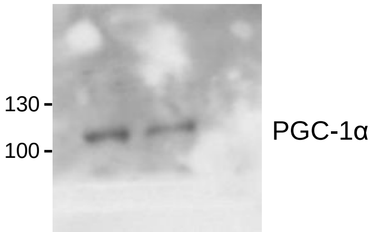


## Fig S5b

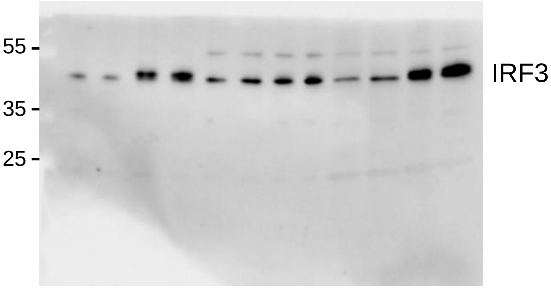


## Fig S5b

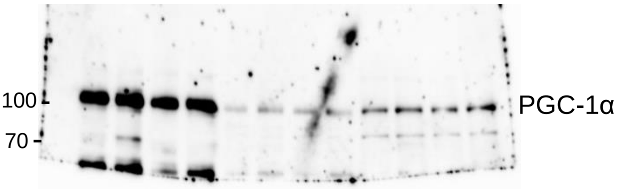


## Fig S5b

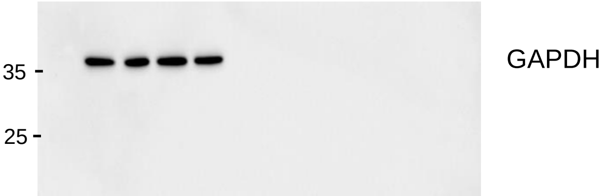


## Fig S5b

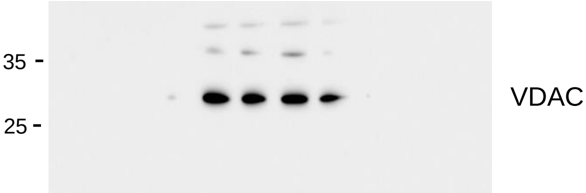


## Fig S5b

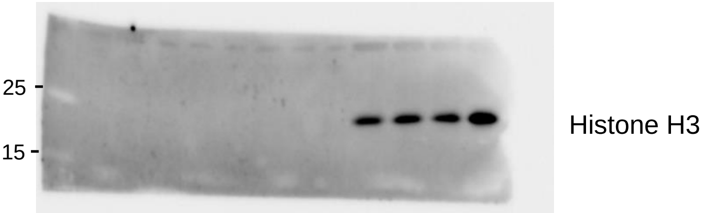


## Fig S5d

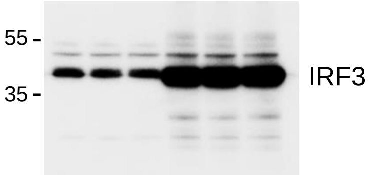


## Fig S5d

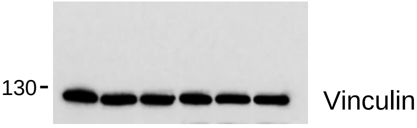

Supplement: Supplementary file 4 — Supplementary Data 2 [file 41467_2026_69792_MOESM4_ESM.pdf]
