# Supplementary material for: Activation of IRF3 in cardiomyocytes impairs mitochondrial oxidative function through PGC-1α inhibition and drives heart failure
Source: Nat Commun. 2026 Feb 27;17:2051. doi: 10.1038/s41467-026-69792-4 (PMC12948977; doi:10.1038/s41467-026-69792-4)
Supplement: Supplementary file 5 — Reporting summary [file 41467_2026_69792_MOESM5_ESM.pdf]

nature portfolio

Corresponding author(s): DAPR_NCOMMS-24-80595B

Last updated by author(s): Dec 15, 2025

Reporting Summary

Nature Portfolio wishes to improve the reproducibility of the work that we publish. This form provides structure for consistency and transparency in reporting. For further information on Nature Portfolio policies, see our Editorial Policies and the Editorial Policy Checklist.

Statistics

For all statistical analyses, confirm that the following items are present in the figure legend, table legend, main text, or Methods section.

n/a	Confirmed	
☐	☒	The exact sample size (*n*) for each experimental group/condition, given as a discrete number and unit of measurement
☐	☒	A statement on whether measurements were taken from distinct samples or whether the same sample was measured repeatedly
☐	☒	The statistical test(s) used AND whether they are one- or two-sided<br>*Only common tests should be described solely by name; describe more complex techniques in the Methods section.*
☒	☐	A description of all covariates tested
☐	☒	A description of any assumptions or corrections, such as tests of normality and adjustment for multiple comparisons
☐	☒	A full description of the statistical parameters including central tendency (e.g. means) or other basic estimates (e.g. regression coefficient) AND variation (e.g. standard deviation) or associated estimates of uncertainty (e.g. confidence intervals)
☐	☒	For null hypothesis testing, the test statistic (e.g. *F*, *t*, *r*) with confidence intervals, effect sizes, degrees of freedom and *P* value noted<br>*Give P values as exact values whenever suitable.*
☒	☐	For Bayesian analysis, information on the choice of priors and Markov chain Monte Carlo settings
☒	☐	For hierarchical and complex designs, identification of the appropriate level for tests and full reporting of outcomes
☒	☐	Estimates of effect sizes (e.g. Cohen's *d*, Pearson's *r*), indicating how they were calculated

Our web collection on statistics for biologists contains articles on many of the points above.

Software and code

Policy information about availability of computer code

Data collection	ABI 7900 HT Fast Real time PCR,<br>LightCycler 480 Roche,<br>Visual Sonics Vevo 3100<br>Amersham Imager 600 (GE healthcare),<br>Chemidoc (Biorad),<br>TECAN Spark multimode reader,<br>Multiskan GO Microplate Spectrophotometer,<br>Agilent Seahorse XFE96 Analyser,<br>Ultimate 3000 HPLC,<br>Q-Exactive Plus quadrupole-Orbitrap MS,<br>Leica Microsystems CMS microscope,<br>Zeiss Axioscan 7
Data analysis	GraphPad Prism version 9, Image J, Lipidyzer software (Lipidomics Workflow Manager software version 1.0.5.0 (SCIEX)), Analyst 1.7.2 (AB SCIEX), OS software suite 2.0.0 (AB SCIEX), El-MAVEN, R package CorrectoR, EdgeR

For manuscripts utilizing custom algorithms or software that are central to the research but not yet described in published literature, software must be made available to editors and reviewers. We strongly encourage code deposition in a community repository (e.g. GitHub). See the Nature Portfolio guidelines for submitting code & software for further information.

Data

Policy information about availability of data

All manuscripts must include a data availability statement. This statement should provide the following information, where applicable:

- Accession codes, unique identifiers, or web links for publicly available datasets
- A description of any restrictions on data availability
- For clinical datasets or third party data, please ensure that the statement adheres to our policy

All data and information supporting the findings in this manuscipt are available within the article, as supplemental or source data file. Additional information regarding plasmids generated, validated and used in this study are available from the corresponding author upon reasonable request. The raw RNA-seq dataset generated in this paper is submitted to NCBI Gene Expression Omnibus (GEO) and can be accessed through accession number GSE283352, GSE283353.

Research involving human participants, their data, or biological material

Policy information about studies with human participants or human data. See also policy information about sex, gender (identity/presentation), and sexual orientation and race, ethnicity and racism.

Reporting on sex and gender	The tissue samples processed and reported in this study were selected independent of the sex or gender.
Reporting on race, ethnicity, or other socially relevant groupings	The current manuscript analysis does not involve reporting on race, ethnicity or other socially relevant groupings.
Population characteristics	The study population included male and female subjects aged 27 years and older.
Recruitment	Non-failing specimens were obtained between 1991 and 1999, where, in rare cases, incompatibilities prevented transplantation.
Ethics oversight	All research shown in this study complies with the relevant ethical regulations. Data analysis involving left ventricle tissue were carried out with written informed consent from patients

Note that full information on the approval of the study protocol must also be provided in the manuscript.

Field-specific reporting

Please select the one below that is the best fit for your research. If you are not sure, read the appropriate sections before making your selection.

☒ Life sciences ☐ Behavioural & social sciences ☐ Ecological, evolutionary & environmental sciences

For a reference copy of the document with all sections, see nature.com/documents/nr-reporting-summary-flat.pdf

Life sciences study design

All studies must disclose on these points even when the disclosure is negative.

Sample size	No statistical methods were used to predetermine the sample size. Samples sizes was chosen according to the standard practices in the relevant field.
Data exclusions	No data exclusions.
Replication	All experiments were carried out with atleast three biological replicates. The exact number of biological replicaets (mice, tissue samples, cells) is mentioned in each Figure legend. In vitro overexpression and knock down studies in cardiomyoctes were perfomed at least three times with similar outcome. In accordance to the 3R principle, LAD and AAV experiments in mice were done as per the approved animal protocols and were not technically repeated.
Randomization	Wild type mice used in this study were assigned randomly to the experiment. Transgenic mice were allocated to the respective groups based on their genotyping results.
Blinding	The investigators were blinded for Surgery, Echocardiography, histology, Immunostaining experiments. For other experiments investigators were not blinded due to the neccesity of knowing the treatment or sample/data collection from instruments.

Reporting for specific materials, systems and methods

We require information from authors about some types of materials, experimental systems and methods used in many studies. Here, indicate whether each material, system or method listed is relevant to your study. If you are not sure if a list item applies to your research, read the appropriate section before selecting a response.

Materials & experimental systems

n/a	Involved in the study
☐	☒ Antibodies
☐	☒ Eukaryotic cell lines
☒	☐ Palaeontology and archaeology
☐	☒ Animals and other organisms
☐	☒ Clinical data
☒	☐ Dual use research of concern
☒	☐ Plants

Methods

n/a	Involved in the study
☒	☐ ChIP-seq
☒	☐ Flow cytometry
☒	☐ MRI-based neuroimaging

Antibodies

Antibodies used

IRF3, 4302; CST, 1:1000
pIRF3 [S396], 29047; CST, 1:1000 for WB and 1:200 for IF
Vinculin, 4650; CST, 1:1000
HA-Tag, 3724; CST, 1:1000
VDAC [D73D12], 4661; CST, 1:1000
Histone H3, 4499; CST, 1:2000
OGT, 24083; CST, 1:1000
p-p38MAPK [Thr180/Tyr182] 9211; CST, 1:1000
p38MAPK , 9212; CST, 1:1000
pAMPK [Thr172],2535; CST, 1:1000
AMPK, 2532; CST, 1:1000
pAKT [S473], 4051; CST, 1:1000
AKT, 9272; CST, 1:1000
O-GlcNAc, 9875),CST, 1:1000
OXPHOS, ab110413, Abcam, 1:5000
PGC-1α, ST1202, MerckMillipore, 1:2000
Flag M2 [F1804], Sigma, 1:1000
GAPDH [G8795], Sigma , 1:10000
aActinin, A7732, Sigma, 1:1000
DAPI, 10236276001, Sigma, 1:1000

Validation

The antibodies were validated by the manufacturer
IRF3, 4302; CST, https://www.cellsignal.com/products/primary-antibodies/irf-3-d83b9-rabbit-mab/4302
pIRF3 [S396], 29047; CST, https://www.cellsignal.com/products/primary-antibodies/phospho-irf-3-ser396-d6o1m-rabbit-mab/29047
Vinculin, 4650; CST, https://www.cellsignal.com/products/primary-antibodies/vinculin-antibody/4650
HA-Tag, 3724; CST, https://www.cellsignal.com/products/primary-antibodies/ha-tag-c29f4-rabbit-mab/3724
VDAC [D73D12], 4661; CST,https://www.cellsignal.com/products/primary-antibodies/vdac-d73d12-rabbit-mab/4661
Histone H3, 4499; CST, https://www.cellsignal.com/products/primary-antibodies/histone-h3-d1h2-xp-rabbit-mab/4499
OGT, 24083; CST,https://www.cellsignal.com/products/primary-antibodies/ogt-d1d8q-rabbit-mab/24083
p-p38MAPK [Thr180/Tyr182] 9211; CST, https://www.cellsignal.com/products/primary-antibodies/phospho-p38-mapk-thr180-tyr182-antibody/9211
p38MAPK , 9212; CST, https://www.cellsignal.com/products/primary-antibodies/p38-mapk-antibody/9212
pAMPK [Thr172],2535; CST, https://www.cellsignal.com/products/primary-antibodies/phospho-ampka-thr172-40h9-rabbit-mab/2535
AMPK, 2532; CST, https://www.cellsignal.com/products/primary-antibodies/ampka-antibody/2532
pAKT [S473], 4051; CST, https://www.cellsignal.com/products/primary-antibodies/phospho-akt-ser473-587f11-mouse-mab/4051
AKT, 9272; CST, https://www.cellsignal.com/products/primary-antibodies/akt-antibody/9272
O-GlcNAc, 9875),CST, https://www.cellsignal.com/products/primary-antibodies/o-glcnac-ctd110-6-mouse-mab/9875
OXPHOS, ab110413, Abcam, https://www.abcam.com/en-us/products/panels/total-oxphos-rodent-wb-antibody-cocktail-ab110413?srsltid=AfmBOopEvDevuDigrlXdqyfc0fXho7xuWTah8RSIcPcYBAW_S_Rwfxwt
PGC-1α, ST1202, MerckMillipore, https://www.sigmaaldrich.com/DE/de/product/mm/st1202?mmredirect=1
Flag M2 [F1804], Sigma, https://www.sigmaaldrich.com/DE/de/product/sigma/f1804?srsltid=AfmBOorjuLh7BBgBMUCczXM_SDtA4V0W84uh4QzHVqW1PmLuRbyTUYhb
GAPDH [G8795], Sigma, https://www.sigmaaldrich.com/DE/de/product/sigma/g8795?srsltid=AfmBOooeZVfKInL80NpALQsOg2X0zhQiWc-cqgC40xHzGGpT8b-knYdO
aActinin, A7732, Sigma, https://www.sigmaaldrich.com/DE/de/product/sigma/a7732?srsltid=AfmBOorbYBZYXVNmLaM8-qk9XdckMat0o922b9ZWnnXVcdLgmFVW0LNe
DAPI, 10236276001, Sigma, https://www.sigmaaldrich.com/DE/de/product/roche/10236276001?utm_source=google&utm_medium=cpc&utm_id=12414022935&utm_campaign=%7Bcampaignname%7D&utm_content=117752976706&utm_term=dapi+10236276001&gad_source=1&gad_campaignid=12414022935&gclid=Cj0KCQjw4qHEBhCDARIsALYKFNM6t-YoYNbpCqT_6LDgg5rEwUPMGDD5XkCp4b2ypK608DxI36b8UkMaAmSFEALw_wcB

Eukaryotic cell lines

Policy information about cell lines and Sex and Gender in Research

Cell line source(s)	HEK293T and HEk293A were purchased from ATCC
Authentication	Cells were purchased from ATCC and ATCC provided the authentication.
Mycoplasma contamination	During the production of adenovirus HEK293T and HEK293A were tested negative for mycoplasma. Later mycoplasma test was not done.
Commonly misidentified lines (See ICLAC register)	No misidentified cell lines were used.

Animals and other research organisms

Policy information about studies involving animals; ARRIVE guidelines recommended for reporting animal research, and Sex and Gender in Research

Laboratory animals	IRF3-2D transgenic mice were generated at BIDMC, Harvard University. IRF3-flox mice was generated in collaboration with TIGM, Texas as described in the manuscript. Strains purchased include: C57BL/6J (JAX: 000664), αMHCMCM (JAX: 005657), Mck-Cre (JAX: 006475), αMHC-Cre (JAX: 011038).
Wild animals	This study didnot involve any wild type animals.
Reporting on sex	For cardiomyocytes cell fractionation experiments, cells from both male and female transgenic mice were used. For other experiments only the experiments done in male mice is reported in this manuscript.
Field-collected samples	No field collected samples were used in this study
Ethics oversight	Animal experiments were approved by the Animal Welfare Officers at University Medical Center Hamburg-Eppendorf, Behörde für Gesundheit und Verbraucherschutz (BGV) Hamburg and Ministerium für Landwirtschaft, ländliche Räume, Europa und Verbraucherschutz (MLLEV) Ministerium für Energiewende, Landwirtschaft, Umwelt, Natur und Digitalisierung (MELUND) of the state of Schleswig-Holstein, and were carried out in accordance with institutional ethical guidelines. Mice had ad libitum access to standard chow diet (Altromin, 1329P) and water. Mice were housed under a regular 12h light/12h dark cycle at constant temperature (23°C).

Note that full information on the approval of the study protocol must also be provided in the manuscript.

Clinical data

Policy information about clinical studies

All manuscripts should comply with the ICMJE guidelines for publication of clinical research and a completed CONSORT checklist must be included with all submissions.

Clinical trial registration	Ethics Committee of the Medical Association Hamburg, File No. 523/116/9.7.1991
Study protocol	Study protocol info is available upon reasonable request.
Data collection	Non-failing specimens were obtained between 1991 and 1999, where, in rare cases, incompatibilities prevented transplantation. Patients provided written informed consent.
Outcomes	Left ventricle tissue samples from non-failing and ischemic patients were collected and stored at -80C. In the current study seven samples from non-failing and ten samples from ischemic patients were processed for total RNA and protein isolation as described in the method section.

Plants

Seed stocks	*Report on the source of all seed stocks or other plant material used. If applicable, state the seed stock centre and catalogue number. If plant specimens were collected from the field, describe the collection location, date and sampling procedures.*
Novel plant genotypes	*Describe the methods by which all novel plant genotypes were produced. This includes those generated by transgenic approaches, gene editing, chemical/radiation-based mutagenesis and hybridization. For transgenic lines, describe the transformation method, the number of independent lines analyzed and the generation upon which experiments were performed. For gene-edited lines, describe the editor used, the endogenous sequence targeted for editing, the targeting guide RNA sequence (if applicable) and how the editor was applied.*
Authentication	*Describe any authentication procedures for each seed stock used or novel genotype generated. Describe any experiments used to assess the effect of a mutation and, where applicable, how potential secondary effects (e.g. second site T-DNA insertions, mosiacism, off-target gene editing) were examined.*
